# Built and natural environment correlates of physical activity of adults living in rural areas: a systematic review

**DOI:** 10.1186/s12966-024-01598-3

**Published:** 2024-05-03

**Authors:** Christina Müller, Lisa Paulsen, Jens Bucksch, Birgit Wallmann-Sperlich

**Affiliations:** 1 Institute of Sport Science, University of Würzburg, Judenbühlweg 11, 97082 Würzburg, Germany; 2 Department of Prevention and Health Promotion, Heidelberg University of Education, Keplerstraße 87, 69120 Heidelberg, Germany

**Keywords:** Adult, Built environment, Natural environment, Physical activity, Rural

## Abstract

**Background:**

According to social-ecological models, the built and natural environment has the potential to facilitate or hinder physical activity (PA). While this potential is well researched in urban areas, a current systematic review of how the built and natural environment is related to PA in rural areas is lacking.

**Methods:**

We searched five databases and included studies for adults (18–65 years) living in rural areas. We included quantitative studies investigating the association between any self-reported or objectively measured characteristic of the built or natural environment and any type of self-reported or objectively measured PA, and qualitative studies that reported on features of the built or natural environment perceived as barriers to or facilitators of PA by the participants. Screening for eligibility and quality assessment (using the Standard Quality Assessment Criteria for Evaluating Primary Research Papers from a Variety of Fields) were done in duplicate. We used a narrative approach to synthesize the results.

**Results:**

Of 2432 non-duplicate records, 51 quantitative and 19 qualitative studies were included. Convincing positive relationships were found between the availability and accessibility of places for exercise and recreation and leisure-time PA as well as between the overall environment and leisure-time PA. Possible positive associations were found between the overall environment and total and transport-related PA, between greenness/natural environment and total PA, between cycling infrastructure and aesthetics and MVPA, and between pedestrian infrastructure and total walking. A possible negative relationship was found between safety and security and total walking. Qualitative studies complemented several environmental facilitators (facilities for exercise and recreation, sidewalks or streets with low traffic, attractive natural environment) and barriers (lack of facilities and destinations, lack of sidewalks, speeding traffic and high traffic volumes, lack of street lighting).

**Conclusions:**

Research investigating the relationship between the built and natural environment and PA behaviors of adults living in rural areas is still limited and there is a need for more high-quality and longitudinal studies. However, our most positive findings indicate that investing in places for exercise and recreation, a safe infrastructure for active transport, and nature-based activities are possible strategies that should be considered to address low levels of PA in rural adults.

**Trial registration:**

PROSPERO: CRD42021283508.

**Supplementary Information:**

The online version contains supplementary material available at 10.1186/s12966-024-01598-3.

## Background

There is convincing evidence that physical activity (PA) contributes substantially to human health and well-being [1, 2]. Regular PA reduces the risk of numerous diseases, such as type 2 diabetes, cardiovascular diseases, and cancer, and improves mental health outcomes [3–6]. However, more than a quarter of adults worldwide are insufficiently active according to the World Health Organization (WHO) recommendations for aerobic PA required to offer health benefits and mitigate health risks [7]. It is therefore important to understand what factors influence PA in different population groups so that measures and interventions can be directed to promote PA.

Social-ecological models are well established to explain PA behaviors [8–10]. According to these models, a multitude of factors on different levels (i.e., intrapersonal, social, cultural, physical, information, and policy environment) influence PA behaviors. Over the past decades, there has been increasing interest in studying associations between the built and natural environment and PA [11]. From a public health perspective, the environment has the potential to affect health and health-related behavior change of the whole population [10]. The built environment refers to any human-made or human-modified features of the physical environment (e.g., buildings, transportation systems, design features, etc.), while the natural environment encompasses any natural features of the physical environment (e.g., trees, grass, water, hilliness etc.) [12–14]. A recently published overview of systematic reviews from high-income countries (according to the World Bank classification [15]) investigating the associations between built environments and PA in different domains (i.e., leisure, transportation, occupation) summarized that there is moderate to high certainty of evidence for positive associations between environments that support active transportation (e.g., walkability, walking infrastructure, street connectivity, land use mix) and transport-related PA among adults [13]. Lower certainty of evidence suggests that leisure, transportation, and total PA are associated with aesthetics, forests/trees, parks, and greenspace/open space [13]. A systematic review of studies across all age groups conducted in low- and middle-income countries (according to the World Bank classification [15]) found that land use mix was positively associated with transport-related PA and the presence of recreation facilities increased leisure-time PA [16].

Most of the current evidence is based on studies in urban areas. However, between 18% (Northern America) and 57% (Africa) of the global population currently live in rural areas [17]. Rural areas are typically defined as territories not included within urban areas (e.g., by the U.S. Bureau of Census [18]). Globally, there is a great variety of criteria to distinguish rural from urban areas, including administrative designations, population size/density, and economic characteristics [17]. Some reviews have shown that the degree of urbanization is associated with adults’ PA levels [13, 19, 20]. Adults living in more urbanized areas tend to walk and cycle more for transport purposes; associations with leisure-time PA are null or negative, and associations with total PA are mixed [13, 20]. To create equal opportunities for healthy and active living, it is important to examine if the environmental characteristics associated with PA in urban populations are relevant to those living in rural areas. A review by Frost et al. summarized the influence of the built environment on the PA of adults living in rural areas [21]. The review concluded that research on this topic was limited. However, the results suggested that associations between elements of the built environment and PA among adults differ between rural and urban areas. The authors included 20 studies published between 2000 and 2008 [21]. Another literature review focusing on the United States confirmed that differences between urban and rural areas as well as rural-specific barriers to PA (e.g., long travel distances, a lack of public transport, and a lack of sidewalks and streetlights) seem to exist [22]. The need for more rural-specific evidence has been stated by both an American and a Canadian “call to action”, since the specific characteristics and challenges of rural areas have been neglected by active living research, policy, and practice for decades [23, 24]. It is important to better understand the factors that facilitate or hinder PA in rural areas to develop and empirically test strategies with a rural-specific theoretical foundation.

During the past 15 years, further studies have investigated associations between elements of the built or natural environment and PA of adults in rural settings. Considering this development, it is worthwhile and timely to systematically re-examine the current evidence base. Therefore, this systematic review aims to identify which elements of the built and natural environment are associated with PA in adults living in rural areas worldwide to gain a current overview of the evidence and to cover a broad spectrum of diverse rural areas. Since there is no internationally recognized definition of a rural area, we incorporate any area described as rural by the authors. To elucidate the full picture of built and natural environmental correlates we focus on quantitative and qualitative studies. While quantitative studies show the relationship between environmental variables and PA outcomes, qualitative studies can add an in-depth understanding of how individuals experience different rural environments [25, 26].

## Methods

This systematic review followed the Preferred Reported Items for Systematic Reviews and Meta-analyses (PRISMA) recommendations [27]. It was prospectively registered at PROSPERO (CRD42021283508).

### Data sources and search strategy

We systematically searched the following electronic databases in October 2021: PUBMED, PsycInfo, Web of Science, TRID, and Engineering Village – GEOBASE & GeoRef. The searches combined the keywords presented in Table 1 covering rural areas, built and natural environments, physical activity, and associations. For rural areas, we used the keyword “rural”. We excluded other possible keywords (village*, “small town”, “small towns”, countryside) based on a sensitivity analysis conducted in PUBMED. These keywords did not identify any additional records without the keyword “rural” in the title or abstract. The results of the sensitivity analysis as well as the full search strategy used for each database are outlined in the supplementary material [see Additional file 1]. Full updated searches were conducted in February 2023. In addition, we screened reference lists from previous reviews [28, 29, 21, 22, 24, 30–32, 23].

**Table 1:** Keywords used in the search strategy

Construct	Keywords
Rural areas	Rural
Built and natural environment	Built environment, physical environment, natural environment, area-level, walkability, bikeability, land use, green space, open space, greenness, blue space, forest, landscape, vegetation, nature, neighborhood, ecological, infrastructure, recreation facilities, sidewalk, park, physical attributes, physical characteristics, playground
Physical activity	Physical activity, sport, exercise, walking, walk, cycling, cycle, bicycle, biking, active transport, active travel, active commuting, everyday activities, motorized travel, motorized transport
Associations	Determinant, correlate, influence, association, predictor, barrier, enabler, facilitator

### Eligibility criteria

We included quantitative and qualitative studies. A quantitative study was eligible for inclusion if it (1) was published in English, (2) included a sample or subsample of adults between 18 and 65 years of age living in rural areas (based on the definition given by the authors), (3) investigated the association between any self-reported or objectively measured characteristic of the built or natural environment and any type of self-reported or objectively measured PA (e.g., total PA, moderate-to-vigorous physical activity (MVPA), walking, cycling). We excluded studies with work-related PA or sedentary behavior as sole outcomes. For this review, we defined the built and natural environment as any natural or human-made features of the physical environment, including land surfaces, vegetation, buildings, and infrastructures [14]. We did not include features of the social environment (e.g., crime-related safety) and features like air quality, noise, weather, or climate. We also excluded studies investigating the association between PA and rurality or the degree of urbanization in general.

A qualitative study was eligible for inclusion if it (1) was published in English, (2) included a sample or subsample of adults between 18 and 65 years of age living in rural areas (based on the definition given by the authors), (3) included at least one qualitative data collection method (e.g., qualitative interviews or focus groups), and (4) reported on or discussed features of the built or natural environment perceived as barriers to or facilitators of PA by the participants.

We excluded any qualitative or quantitative study with a sample including exclusively or predominantly (more than 50% of the sample) children, adolescents, or older adults (≥ 65 years), including exclusively urban populations, or reporting only combined results for urban and rural populations.

### Study selection

The citations and abstracts of all identified records were imported into the web-based Covidence systematic review tool, and all duplicates were removed. Two reviewers independently screened the titles and abstracts of the records for inclusion against eligibility criteria. Any disagreements were resolved through discussion involving a third reviewer. Full-text articles were retrieved if the information provided in the title and abstract met the inclusion criteria or if eligibility was uncertain. Two reviewers independently screened the full texts of all potentially relevant records. In cases of conflict, a third reviewer was involved.

### Data extraction and quality assessment

The data extraction of 40% of the studies (*n* = 29) was done independently by two persons (CM + one co-author or one trained student). Any discrepancies were resolved involving two co-authors (BWS, JB). The remaining 60% of the studies (*n* = 41) were extracted by only one author (CM), but in any case of uncertainty, a second or third person (BWS, JB) was involved. The following information was extracted (if possible) using standardized forms: lead author, year, title, the country in which the study was conducted, the aim of the study, study design, definition of rurality, setting, priority population, the total number of participants, percentage of female participants, mean age (standard deviation), age range, types of built/natural environment assessed, measurement instrument(s) used to assess the built/natural environment (subjective/objective), physical activity outcome(s) assessed, measurement instrument(s) used to assess the physical activity outcome(s) (subjective/objective), significant quantitative results, non-significant quantitative results, and qualitative results.

Two reviewers (CM and a second reviewer) independently completed the quality assessment for all studies. We used the Standard Quality Assessment Criteria for Evaluating Primary Research Papers from a Variety of Fields [33], as the criteria can be applied to diverse qualitative and quantitative study designs. We applied the checklist for quantitative studies with 14 items and the checklist for qualitative studies with 10 items, which were all scored depending on the degree to which they were met or reported (yes = 2, partial = 1, no = 0). Items not applicable to a particular study were marked “N/A” and excluded when calculating the summary score. Any disagreements were resolved through discussion involving a third reviewer. A summary score was calculated for each study expressed as a percentage (with 100% the best possible quality). We did not exclude any studies from the review based on quality.

### Synthesis of results

We used a narrative approach to synthesize the results of both quantitative and qualitative studies. Due to the heterogeneity of measures of the built/natural environment and PA, a meta-analysis of the quantitative studies was not reasonable. We extracted all individual associations from each quantitative study and coded each correlate using categories previously described in the built and natural environment literature: availability and accessibility of destinations [14], availability and accessibility of places for exercise or recreation [34], availability and accessibility of public transport [35, 36], overall accessibility [14, 36], density [35, 36], land use [36, 37], connectivity [36, 37], pedestrian infrastructure [36], cycling infrastructure [38], safety and security [36, 39], aesthetics [36], greenness/natural environment [12], hilliness [20], and overall environment [39]. The definitions of the categories and their expected associations with PA are presented in Table 2. The results were additionally stratified by type of PA. When available, only the most adjusted effect estimates (e.g., odds ratios adjusted for possible covariates like age, gender, or health status instead of crude odds ratios) were reported to reduce potential bias. Associations were classified as positive, negative, or non-significant. We summarized the results by applying a method of evidence coding adapted from a previous systematic review of Wang and Wen (Table 3) [40]. The degree of the relationship between a built or natural environment factor and a type of PA was coded as convincing, possible, or inconclusive, depending on the proportion of studies concluding the same direction (see Table 3). Summary results were only given to associations investigated in at least four studies to ensure an adequate foundation for conclusions and to increase the reliability of the results by avoiding overgeneralization. Studies can appear in more than one category of association (positive, negative, non-significant) if they reach more than one conclusion (e.g., for different sub-samples or different buffers).

**Table 2:** Definition of categories used in the synthesis of results

Category	Definition
Availability and accessibility of destinations	Presence of or ease of access to places offering some type of service or goods where people go with a purpose, such as shops, churches, schools, workplaces, etc. Destinations within walking or cycling distance are expected to increase active travel and thus PA [14].
Availability and accessibility of places for exercise or recreation	Presence of or ease of access to indoor and outdoor spaces and facilities designed for exercise or leisure activities, including parks, swimming pools, walking trails, fitness centers, etc.; these places provide opportunities for leisure-time PA and are therefore expected to be positively associated with PA [34].
Availability and accessibility of public transport	Distance to or density of public transport stops (e.g., railway station or bus stop); a shorter distance to as well as a higher number or density of public transport stops is expected to increase walking and thus PA [35, 36]
Overall accessibility	Presence of or ease of access to places or public transport; better accessibility means shorter distances and shorter distances are expected to increase active travel and thus PA [14, 36].
Density	Population or dwelling units per unit area [35]; high residential or population density is expected to be positively associated with walking and PA, as it reduces distance and time of travel between residences and destinations [36].
Land use	Type of use of physical space within a given area (e.g., residential, commercial, industrial, agricultural); mixed land use providing nonresidential destinations is expected to be associated with more walking and PA [36, 37].
Connectivity	Characteristics of the street design that facilitate direct travel between two points, such as a high intersection density, alternative routes, and more street integration; higher connectivity is expected to increase walking and cycling by providing more potential routes and shorter distances to destinations [36, 37].
Pedestrian infrastructure	The presence and quality of sidewalks (including maintenance, width, absence of obstructions) are expected to be positively correlated with walking [36].
Cycling infrastructure	The presence and quality of bicycle lanes/paths (including maintenance, width, absence of obstructions) or bicycle-friendly streets are expected to be positively correlated with cycling [38].
Safety and security	Safety refers to pedestrians and cyclists being protected from motorized traffic by low traffic volumes or safety and traffic calming infrastructure (e.g., buffers, crosswalks); security refers to pedestrians being protected from crime and incivilities, mostly by street lighting; higher safety and security are expected to be associated with higher walking, cycling, and PA [36, 39].
Aesthetics	Presence of interesting sights, maintenance, cleanliness, and absence of physical disorder; aesthetics is expected to be positively associated with leisure-time PA and walking [36].
Greenness/ natural environment	Elements of nature, such as trees, grass, plants or water, or the greenness of an environment are expected to be positively associated with outdoor leisure-time PA and walking [12].
Hilliness	Hilliness/an increased slope makes walking and cycling more difficult and is therefore expected to be negatively associated with transport-related PA [20].
Overall environment	An overall rating of how the physical environment enables or hinders PA; often multidimensional and combines the dimensions described above into an index or overall score [39].

We extracted all reported themes that could be classified as characteristics of the built or natural environment from the included qualitative studies and classified them as barriers or facilitators. The categorization displayed in Table 2 was then applied to the themes. Since the qualitative studies described interactions between correlates from different categories, some categories were combined in the presentation of the results.

**Table 3:** Criteria for coding summary results [adapted from Wang and Wen [40]]

Type of result	Number of studies and relative agreement	Code
Not able to get a summary result	0–3 studies	N/A
Convincing positive relationship	≥ 4 studies, positive relationship found in ≥ 60% of studies and negative relationship in < 25% of studies	+ +
Possible positive relationship	≥ 4 studies, positive relationship found in 41–60% of studies and negative relationship in < 25% of studies	+
Convincingly not related	≥ 4 studies, no significant relationship found in ≥ 60% of studies and any other direction in < 40% of studies	0 0
Possible negative relationship	≥ 4 studies, negative relationship found in 41–60% of studies and positive relationship in < 25% of studies	-
Convincing negative relationship	≥ 4 studies, negative relationship found in ≥ 60% of studies and positive relationship in < 25% of studies	- -
Inconclusive	≥ 4 studies, no consistent associations	?

## Results

**Fig. 1:**
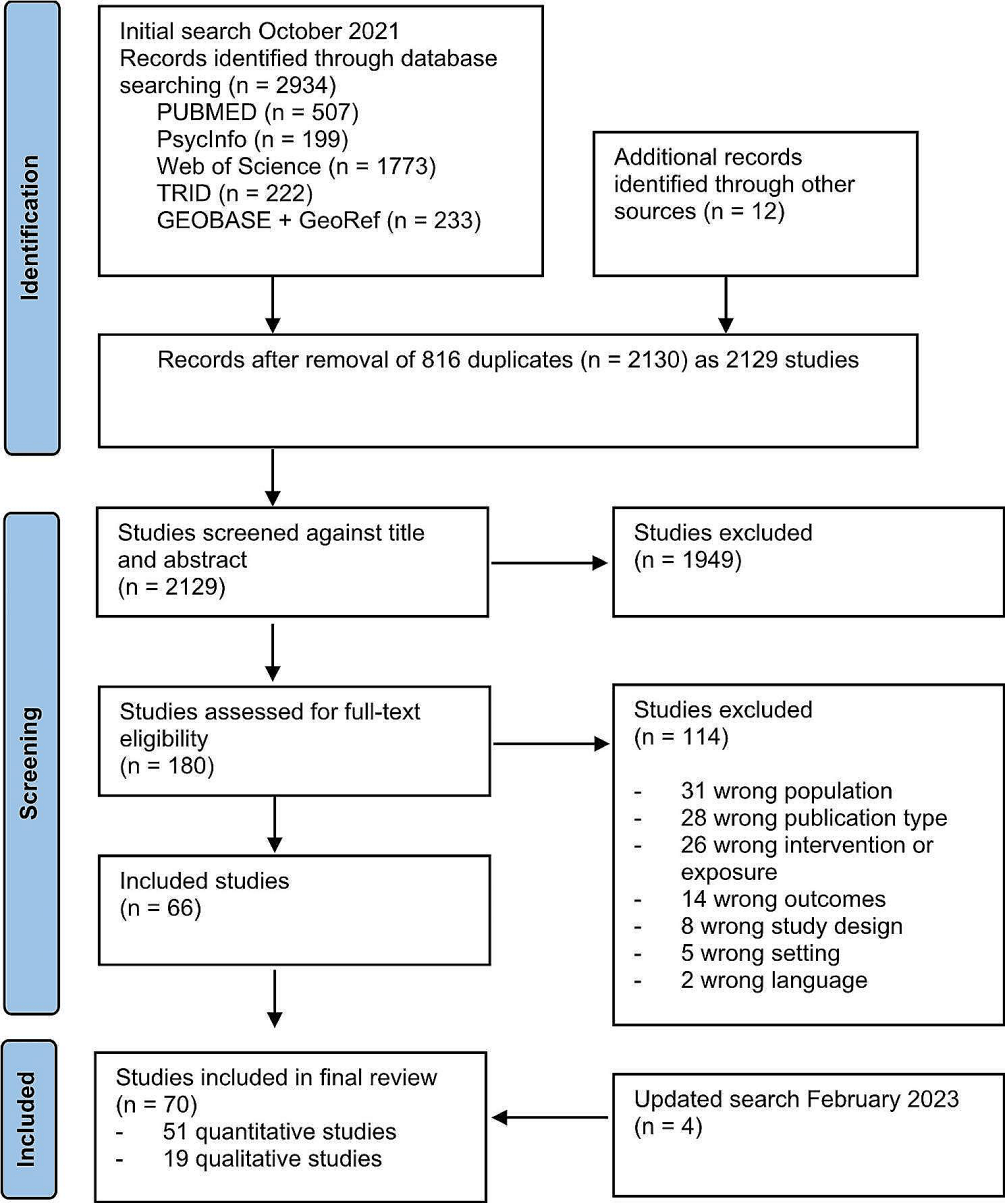
PRISMA flowchart of the study selection process

Figure 1 presents the flow diagram of included and excluded articles. The initial search resulted in 2130 potential articles, yielding 2129 individual studies since one study was published in two articles. Of these, we fully read 180 and included 66 studies in our analysis. After the exclusion of duplicates, the second search in February 2023 resulted in 303 potential articles. We fully read 24 articles and included a further four studies in our analysis. In sum, we included 70 studies in our analysis.

### Characteristics of quantitative studies

Characteristics of the included quantitative studies (*n* = 51) are presented in Table 4. The quantitative studies comprised 47 cross-sectional studies with individuals as the unit of analysis (92.2%) and four ecological studies with groups of people as the unit of analysis (7.8%). Most studies (*n* = 34; 66.7%) were conducted in the USA and focused on adults of all ages (*n* = 20; 39.2%) and genders (*n* = 43; 84.3%). Two studies (3.9%) included only inactive persons [41, 42], and one study (2.0%) only individuals with diabetes [43]. Sample sizes (excluding participants from urban areas) ranged from 143 [44] to 473,296 [45] (median: 585). 24 studies (47.1%) did not report the applied definition of rural areas, 11 studies (21.6%) applied a definition of rural areas by the U.S. Bureau of Census. All reported definitions of rural areas are described in the supplementary material (additional file 3). All but six studies (88.2%) relied solely on self-reported PA measures and MVPA was the most frequent type of PA (*n* = 21; 41.2%). The environmental characteristics investigated most frequently were the availability and accessibility of places for exercise and recreation (*n* = 27; 52.9%) and safety and security (*n* = 26; 51.0%). The quality scores of the included quantitative studies ranged from 55% [46] to 100% [47–53], with a mean score of 84%.

**Table 4:** Characteristics of quantitative studies (*n* = 51)

Characteristic	No.	%	References
**Location**			
USA	34	66.7%	[52, 54–56, 44, 43, 57–64, 48, 65, 66, 53, 67–69, 41, 45, 70–80]
Australia	4	7.8%	[42, 49–51]
China	4	7.8%	[81–83, 47]
India	2	3.9%	[46, 84]
Japan	2	3.9%	[85, 86]
Other (Canada, Germany, Korea, Norway, South Africa)	5	9.8%	[87–91]
**Sample size (rural only)**			
101–300	10	19.6%	[41–44, 61, 69, 75, 76, 83, 88]
301–500	10	19.6%	[56, 62, 64, 65, 68, 71, 82, 85, 89, 91]
501–1000	8	15.7%	[51, 53, 58, 66, 73, 74, 86, 90]
1001–2500	12	23.5%	[48–50, 54, 55, 57, 63, 67, 70, 72, 80, 81]
> 2500	7	13.7%	[87, 47, 45, 77–79, 46]
N/A (aggregated data)	4	7.8%	[52, 59, 60, 84]
**Age**			
Young + middle-aged (18–65)	5	9.8%	[49, 58, 67, 74, 87]
Middle-aged only (40–65)	2	3.9%	[50, 85]
Middle-aged + older (35+)	8	15.7%	[41, 42, 66, 73, 77, 80, 86, 89]
All age groups	20	39.2%	[43–45, 47, 48, 54–56, 62, 64, 70, 72, 75, 78, 79, 81–83, 88, 91]
Age range not reported	16	31.4%	[52, 51, 57, 59–61, 63, 65, 53, 68, 69, 71, 84, 90, 76, 46]
**Gender**			
Women only	8	15.7%	[41, 49, 58, 73, 74, 77, 80, 85]
Mixed	43	84.3%	All others
**Types of PA**			
Unspecified/total PA	9	17.6%	[42, 44, 46, 50, 63, 65, 77, 89, 90]
MVPA	21	41.2%	[41, 44, 48, 52, 54, 55, 58, 61, 64, 66, 70–75, 80, 85, 87, 90, 91]
Leisure-time PA	10	19.6%	[43, 45, 49, 50, 53, 55, 63, 83, 88, 89]
Sports/exercise	2	3.9%	[47, 81]
Transport-related PA	7	13.7%	[44, 49, 50, 55, 62, 63, 89]
Active commuting	1	2.0%	[61]
Total walking	9	17.6%	[48, 51, 54, 65, 67, 68, 72, 88, 91]
Leisure/recreational walking	5	9.8%	[69, 76, 78, 79, 89]
Walking for transport	7	13.7%	[57, 69, 76, 78, 79, 84, 86]
Walking for commuting purposes	3	5.9%	[59, 60, 86]
Cycling for commuting purposes	3	5.9%	[59, 60, 84]
Total walking and cycling	1	2.0%	[56]
Car use (vs. walking)	1	2.0%	[82]
**PA measures**			
**Device-based**			
Accelerometers	4	7.8%	[41, 75, 76, 90]
Pedometers	2	3.9%	[42, 65]
**Self-reported**			
IPAQ	14	27.5%	[44, 49, 50, 57, 62, 63, 85, 66, 87, 67–69, 89, 91]
BRFSS	11	21.6%	[43, 45, 48, 52–54, 61, 67, 70, 72, 73]
GPAQ	2	3.9%	[55, 83]
Other questionnaires	21	41.2%	[81, 82, 51, 56, 58–60, 64, 65, 44, 88, 86, 47, 71, 74, 84, 46, 77–80]
**Environmental characteristics**			
Availability and accessibility of destinations	15	29.4%	[41, 43, 57, 58, 61, 62, 67, 74, 76, 79, 82, 85, 88, 89, 91]
Availability and accessibility of places for exercise or recreation	27	52.9%	[52, 81, 55, 43, 57–62, 64, 48, 65, 85, 53, 87, 41, 45, 70, 71, 74, 73, 75, 76, 91, 79, 80]
Availability and accessibility of public transport	9	17.6%	[57, 61, 67, 79, 82, 84, 85, 89, 91]
Overall accessibility	6	11.8%	[51, 63, 68, 69, 82, 90]
Density	7	13.7%	[46, 60, 61, 76, 82, 85, 91]
Land use	4	7.8%	[57, 63, 67, 76]
Connectivity	8	15.7%	[46, 59, 60, 76, 82, 86, 89, 91]
Pedestrian infrastructure	17	33.3%	[41, 43, 44, 54, 58, 61, 62, 64, 67, 72, 74, 76, 79, 80, 85, 89, 91]
Cycling infrastructure	6	11.8%	[41, 61, 62, 85, 87, 91]
Safety and security	26	51.0%	[54, 55, 44, 43, 83, 57, 42, 58, 61–63, 48, 85, 88, 67–69, 41, 89, 70, 74–76, 46, 91, 80]
Aesthetics	16	31.4%	[49, 50, 43, 83, 61–63, 85, 88, 68, 69, 47, 41, 89, 91, 80]
Greenness/natural environment	10	19.6%	[41, 43–46, 59, 60, 76, 77, 89]
Hilliness	5	9.8%	[44, 46, 67, 76, 80]
Overall environment	20	39.2%	[82, 55, 51, 56, 44, 49, 50, 43, 42, 62–66, 53, 41, 75, 90, 78, 79]
**Environment measures**			
**Geographic information systems (GIS) (objective)**	15	29.4%	[41, 45, 46, 52, 57, 59, 60, 67, 76–78, 82, 85–87]
**Environmental audits (objective)**	3	5.9%	[62, 63, 65]
**Questionnaires (subjective)**			
NEWS	7	13.7%	[51, 55, 57, 67, 75, 83, 89]
RALPESS	5	9.8%	[53, 55, 64, 67, 75]
IPAQ-E	3	5.9%	[61, 85, 91]
Other questionnaires	29	56.9%	[54, 81, 82, 56, 44, 49, 50, 43, 42, 58, 62, 48, 65, 66, 88, 68, 69, 47, 41, 70–74, 84, 90, 76, 79, 80]
**Quality assessment**			
Very high (100%)	7	13.7%	[47–53]
High (85–99%)	21	41.2%	[42, 45, 55, 59, 60, 62, 66, 68, 70, 72, 74, 75, 79, 81–87, 90]
Medium (60–84%)	21	41.2%	[58, 61, 63, 65, 54, 57, 43, 56, 64, 88, 73, 91, 67, 69, 41, 76–78, 80, 71, 89]
Low (< 60%)	2	3.9%	[44, 46]

### Characteristics of qualitative studies

Table 5 provides an overview of the qualitative studies (*n* = 19); two of them (10.5%) were mixed-methods studies of which only the qualitative part was included in the review, as the quantitative parts were analyzed only descriptively [95, 96]. 89.5% of the studies (*n* = 17) were conducted in the USA. Ten studies (52.6%) did not report the applied definition of rural areas. The definitions applied in the other studies are summarized in the supplementary material (additional file 3). Age range was not reported in eight studies (42.1%), six studies (31.6%) focused on adults between 18 and 65 years of age, the remaining on adults of all ages (*n* = 3; 15.8%) or middle-aged and older adults (*n* = 2; 10.5%). Eight studies (42.1%) included only women [97–104], five studies (26.3%) only inactive persons [98–101, 105], and one study (5.3%) only patients with type-2 diabetes attending a tertiary care facility (diabetes clinic) [106]. Two studies (10.5%) focused on American Indian adults living in reservation communities [101, 107], one study (5.3%) on Latina immigrants [98], one study (5.3%) on persons with low incomes [108], and one study (5.3%) on women with low incomes who were the primary caretaker of at least one child [102]. Focus groups were the most common data collection approach, used in 13 (68.4%) of the studies [95–101, 103–105, 108–110]. There was a diversity of analytical approaches. However, only six studies provided a clearly described and systematic data analysis [95, 105, 107, 108, 110, 111]. Eleven studies (57.9%) used one or more verification procedures to establish credibility, such as triangulation, prolonged engagement in the field, or inter-rater reliability [95–97, 101, 103–105, 108–110, 112]. The criterion “reflexivity of the account” [33] was fully fulfilled in only one study [107], meaning that the authors explicitly assessed the likely impact of the researchers’ characteristics (such as age, sex, and professional status) and the methods used on the data obtained [33], and partly fulfilled in two studies [96, 97]. Overall, the quality scores ranged from 30% [99] to 85% [105, 108], with a mean score of 64%.

**Table 5:** Characteristics of qualitative studies (*n* = 19)

Characteristic	No.	%	References
**Location**			
USA	17	89.5%	[95–105, 107–111, 113]
Australia	1	5.3%	[112]
Sri Lanka	1	5.3%	[106]
**Sample size (rural only)**			
10–30	6	31.6%	[101, 103, 107–110]
31–50	5	26.3%	[95, 98, 99, 106, 112]
51–100	7	36.8%	[96, 97, 100, 102, 104, 111, 113]
> 100	1	5.3%	[105]
**Age**			
Young + middle-aged (18–65)	6	31.6%	[98–101, 104, 112]
Middle-aged + older (40+)	2	10.5%	[105, 111]
All age groups	3	15.8%	[106, 109, 110]
Age range not reported	8	42.1%	[95–97, 102, 103, 107, 108, 113]
**Gender**			
Women only	8	42.1%	[97–104]
Mixed	11	57.9%	[95, 96, 105–113]
**Data collection instrument**			
Focus groups	13	68.4%	[95–101, 103–105, 108–110]
Interviews	6	31.6%	[102, 106, 110–113]
Photovoice	1	5.3%	[110]
Nominal Group Technique	1	5.3%	[107]
**Analytical approach**			
Content analysis	2	10.5%	[97, 111]
Thematic analysis	2	10.5%	[105, 112]
Grounded theory approach	2	10.5%	[95, 102]
Framework approach	1	5.3%	[106]
Phenomenological techniques	1	5.3%	[104]
Explanation building	1	5.3%	[103]
Nominal Group Technique	1	5.3%	[107]
**Quality assessment**			
High (85%)	2	10.5%	[105, 108]
Medium (60–84%)	10	52.6%	[95, 98, 101, 103, 104, 107, 109–112]
Low (< 60%)	7	36.8%	[96, 97, 99, 100, 102, 106, 113]

### Synthesis of quantitative studies

A summary of the synthesis of the included quantitative studies is displayed in Table 6. Overall, we found two convincing and six possible positive relationships. The following section summarizes the results for each environmental characteristic stratified by type of PA. Only combinations of environmental characteristics and types of PA that were investigated in at least four studies are reported. For the availability and accessibility of public transport, the overall accessibility, density, land use, connectivity, and hilliness, each type of PA was investigated in less than four studies, so no summary results could be derived. The detailed results for each environmental characteristic are shown in the supplementary tables [see Additional file 2].

#### Availability and accessibility of destinations

15 studies examined the relationship between the availability and accessibility of destinations and PA. MVPA was investigated in six studies (two of high and four of medium quality) and was consistently not associated with the availability and accessibility of destinations [41, 58, 61, 74, 85, 91]. Any other type of PA was investigated in less than four studies, so no summary results could be derived. Across all types of PA, positive associations were reported in six studies, negative associations were reported in two studies, and non-significant associations were reported in 14 studies [see Additional file 2].

#### Availability and accessibility of places for exercise and recreation

Twenty-seven studies examined the relationship between the availability and accessibility of places for exercise and recreation and PA. A convincing positive relationship (3/4 studies) was found between the availability and accessibility of places for exercise and recreation and leisure-time PA. Beck et al. found a positive association between perceived access to indoor recreation facilities (RALPESS) and self-reported leisure-time PA (GPAQ) in a high-quality study [55]. Deshpande et al. reported that perceived longer distances to fitness clubs, parks, recreation centers, walking trails, and schools that allow the public to use their facilities for PA are associated with less self-reported leisure-time PA (BRFSS) [43]. In the same study, which was of medium quality, the distance to public swimming pools and the item “many places for PA (not including walking)” were not associated with leisure-time PA [43]. Michimi et al. conducted a high-quality study and found a significant positive association between an objective county-level recreational opportunity index and self-reported leisure-time PA (BRFSS) [45]. Kegler et al. found no associations between indoor exercise areas, organizational facilities, and outdoor exercise areas (RALPESS) and self-reported leisure-time PA (BRFSS) in a study of very high quality [53]. MVPA was examined in 16 studies and convincingly not related to the availability and accessibility of places for exercise and recreation [see Additional file 2]. However, the two studies using objective measures of the recreation environment, both with (very) high quality, found positive associations between a recreation environment index [52], the number of hiking trails [87], and the number of sports parks [87] and self-reported MVPA. The number of exercise facilities and the number of parks were not related to self-reported MVPA in one study [87]. Any other type of PA was investigated in less than four studies, so no summary results could be derived. Across all types of PA, positive associations were reported in 12 studies, a negative association was reported in one study, and non-significant associations were reported in 24 studies [see Additional file 2].

#### Pedestrian infrastructure

Seventeen studies examined the relationship between pedestrian infrastructure and PA. All but one study [62] used subjective measures of the pedestrian environment. The pedestrian infrastructure was consistently unrelated to MVPA, with only one out of eleven studies reporting a significant (positive) association [61]. There is a possible positive association with total walking. Addy et al. (medium quality) and Reed et al. (high quality) reported that the presence of neighborhood sidewalks was positively associated with irregular walking (vs. no walking), but not associated with regular walking (vs. no walking) [54, 72]. Two other studies of medium quality found no significant associations between pedestrian infrastructure and total walking [67, 91]. Any other type of PA was investigated in less than four studies, so no summary results could be derived. Across all types of PA, positive associations were reported in six studies and non-significant associations were reported in all 17 studies. No negative associations were reported.

#### Cycling infrastructure

Six studies examined the relationship between cycling infrastructure and PA. There is a possible positive relationship between the cycling infrastructure and MVPA. In a high-quality study, Kim et al. found a significant positive relationship between the objective presence of cycling facilities and MVPA [87]. In a medium-quality study, Wallmann et al. found a positive relationship between the perceived maintenance of places for bicycling and MVPA, but no significant relationship between the perceived presence of cycling facilities and MVPA [91]. In another high-quality study, Kamada et al. found a positive association between the perceived presence of cycling facilities and MVPA only in sufficiently active individuals, but not in those who were insufficiently active [85]. Any other type of PA was investigated in less than four studies, so no summary results could be derived. None of the identified studies examined associations between cycling infrastructure and cycling. Across all types of PA, positive associations were reported in three studies, and non-significant associations were reported in five studies. No negative associations were reported.

#### Safety and security

Twenty-six studies examined the relationship between PA and safety/security features (including street lighting, low speed of traffic, traffic volume, crosswalks/pedestrian signals, pedestrian accident rates, RALA pedestrian safety and physical security scores, perceived overall safety, perceived safety from traffic, shoulders on streets for safe walking, and buffer between sidewalk and street). The investigated safety and security features were convincingly unrelated to MVPA, leisure-time PA, and transport-related PA [see Additional file 2]. There is a possible negative relationship between safety and security and total walking. Wallmann et al. found that the item “There is so much traffic on the streets that it makes it difficult or unpleasant to walk in my neighborhood” was positively associated with total walking [91]. Hooker et al. found a negative relationship between moderate (vs. heavy) traffic in the neighborhood and total walking in white adults, but not in African American adults, and no significant relationship between light (vs. heavy traffic) and total walking in any population [48]. Kirby et al. found a negative relationship between the perceived safety of the community for walking and total walking in Aboriginal adults [88]. Results were inconclusive for total/unspecified PA. Any other type of PA was investigated in less than four studies, so no summary results could be derived. Across all types of PA, positive associations were reported in eight studies, negative associations were reported in seven studies, and non-significant associations were reported in 25 studies.

#### Aesthetics

Sixteen studies examined the relationship between aesthetics and PA. Two studies used audit scores (WASABE, RALA) [62, 63], and the others subjective scales. No negative associations were reported. Aesthetics was convincingly unrelated to leisure-time PA and transport-related PA [see Additional file 2]. For MVPA, there is a possible positive relationship. Kamada et al. found a significant positive relationship between the item “There are many interesting things to look at while walking in my neighborhood” (IPAQ-E) and self-reported MVPA in sufficiently active women, but not in those who were insufficiently active [85]. The same item was not associated with MVPA in three other studies [41, 61, 91]. Lo et al. found a positive association between the item “My community is generally free from garbage, litter, or broken glass” and objectively measured MVPA, but no significant association with the item “My community is well maintained” [41]. Any other type of PA was investigated in less than four studies, so no summary results could be derived. Across all types of PA, positive associations were reported in eight studies and non-significant associations were reported in 15 studies.

#### Greenness/natural environment

Ten studies examined the relationship between greenness or the natural environment and PA. For total/unspecified PA, there is a possible positive relationship. Valson et al. found a positive association between objective greenness in a 1600-m buffer and self-reported PA in Indian adults [46]. Villeneuve et al. found a positive association between the upper tertile of an objective greenness index including forest, shrubland, and herbaceous land covers in a 250-m buffer and a 500-m buffer and self-reported PA in a large sample of US women [77]. The upper tertile of a second greenness index including forest, shrubland, and herbaceous land covers as well as developed open spaces was only associated with self-reported PA in a 250-m buffer, but not in a 500-m buffer [77]. In addition, the percentage of impervious surfaces (such as pavements and rooftops) in a 250-m buffer and a 500-m buffer was negatively related to women’s self-reported PA [77]. Subjective measures of the presence of hunting/conservation areas [44] and trees [89] were not related to self-reported PA. Any other type of PA was investigated in less than four studies, so no summary results could be derived. Across all types of PA, positive associations were reported in four studies, negative associations were reported in two studies, and non-significant associations were reported in six studies.

#### Overall environment

20 Studies examined the relationship between the overall environment and PA. There is a possible positive association with total/unspecified PA, as three out of six studies reported at least one positive association [42, 44, 50], as well as a possible positive association with transport-related PA [44, 49, 50]. Leisure-time PA is convincingly related to the overall environment, as four out of six studies found significant positive associations [43, 50, 53, 63]. Cleland et al. investigated the perceived physical activity environment as the sum of seven items (‘My neighborhood offers many opportunities to be physically active’, ‘Local sports clubs and other facilities in my neighborhood offer many opportunities to get exercise’, ‘It is pleasant to walk in my neighborhood’, ‘The trees in my neighborhood provide enough shade’, ‘In my neighborhood it is easy to walk places’, ‘I often see other people walking in my neighborhood’, and ‘I often see other people exercising (e.g., jogging, bicycling, playing sports) in my neighborhood’) [50]. This score was positively associated with self-reported leisure-time PA in adults aged 55–65 years [50], but not in a sample of women aged 18–45 years [49]. Deshpande et al. investigated the overall rating of the community as a place to be physically active [43]. Gustat et al. found an overall built environment score from the RALA street segment audit tool, combining the categories path features, pedestrian safety features, segment aesthetics, physical security, destinations, and land use, positively associated with self-reported leisure-time PA in a 1.50-mile buffer surrounding the street segment of residence, but not in a 0.00-mile buffer, a 0.25-mile buffer, a 0.50-mile buffer, and a 1.00-mile buffer [63]. In the same study, the path features score was not related to self-reported leisure-time PA in any buffer [63]. Kegler et al. found positive associations between self-reported leisure-time PA and the RALPESS overall perceived physical environment score and the town center connectivity score (“There are shopping areas and places to eat in the town center”; “There are sidewalks in the town center”; “The sidewalks are nice to use in the town center (e.g., they are shaded, there are pleasant things to look at, no trash, well kept)”; “The streets are marked where I should cross in the town center or there are crosswalks”; “The area around the town center has working streetlights”) [53]. They found no significant association between an adapted “area around the home” score and self-reported leisure-time PA [53]. Beck et al. found no association between the “area around the home” score and self-reported leisure-time PA either [55]. Across all types of PA, positive associations were reported in 11 studies, negative associations were reported in one study, and non-significant associations were reported in 16 studies.

**Table 6:** Summary results of evidence on the relationship between environmental factors and different types of PA

	+ +	+	0 0	-	?
Unspecified/ total PA		• Greenness/natural environment • Overall environment			• Safety and security
MVPA		• Cycling infrastructure • Aesthetics	• Availability and accessibility of destinations • Availability and accessibility of places for exercise or recreation • Pedestrian infrastructure • Safety and security • Overall environment		
Leisure-time PA	• Availability and accessibility of places for exercise or recreation • Overall environment		• Safety and security • Aesthetics		
Transport-related PA		• Overall environment	• Safety and security • Aesthetics		
Total walking		• Pedestrian infrastructure		• Safety and security	

+ + convincing positive relationship (≥ 4 studies, positive relationship found in ≥ 60% of studies and negative relationship in < 25% of studies); + possible positive relationship (≥ 4 studies, positive relationship found in 41–60% of studies and negative relationship in < 25% of studies); 0 0 convincingly not related (≥ 4 studies, no significant relationship found in ≥ 60% of studies and any other direction in < 40%); - possible negative relationship (≥ 4 studies, negative relationship found in 41–60% of studies and positive relationship in < 25% of studies); ? inconclusive (≥ 4 studies, no consistent associations); not included: - - convincing negative relationship (no convincing negative relationships were found)

### Synthesis of qualitative studies

The included qualitative studies described several environmental characteristics that the participants considered to facilitate or hinder PA. Since they also described interactions between different characteristics, we combined some of the pre-defined categories.

#### Availability and accessibility of places for exercise and recreation, destinations, and public transport

In almost all studies, including the two studies of high quality [105, 108], the availability and accessibility of places for exercise and recreation was a central theme. Participants identified facilities such as swimming pools, parks, sports fields, walking trails, or public schools as resources for PA [97, 100, 103, 105, 107–110]. Meanwhile, the lack of (diversity of) facilities close to people’s homes was frequently mentioned as a barrier [95–97, 99–102, 104–108, 111, 112]. Some participants described a lack of affordable facilities and the cost of facilities, such as gyms, as a barrier [96, 98, 100, 101, 108, 112]. Inconvenient opening hours and poor maintenance of facilities also prevented some people from using them [95, 100, 101, 105]. In some rural communities, fields or tracks on school property were not available for public use, or facilities like swimming pools were reserved for school teams [95, 98, 104, 108, 109]. In a few studies, participants raised concerns related to the accessibility of trails or other facilities for people with impaired mobility or people with young children [95, 112]. Some studies reported that (not) having destinations nearby influenced how much people walked [96, 98, 104, 109, 110]. The lack of public transport was described as a barrier to PA in two studies [96, 98].

#### Pedestrian and cycling infrastructure, safety, and connectivity

The participants of several studies discussed issues in the pedestrian and cycling infrastructure. Kegler et al. described that some participants appreciated that there was plenty of space for walking and riding bikes in their community; the participants in another study appreciated the availability of a bike path [107, 111]. In the study by Chrisman et al., some participants stated that sidewalks facilitated walking and cycling in their community, while others described using neighborhood streets for walking and cycling because sidewalks were too narrow and walking and cycling on streets was safe as there was not much traffic [109]. The lack of sidewalks was discussed as a barrier to walking in several studies, including the two studies of high quality [98–100, 102, 104, 105, 108, 109]. Some participants also mentioned the poor condition of sidewalks or roads that made walking difficult (uneven pavement, roads that are muddy or have loose gravel, not adequately cleared from snow in winter) [99, 101, 103–106, 108, 109]. Low-traffic roads were seen as encouraging to walkers [100, 109, 111]. On the other hand, high traffic volumes and speeding traffic were often described as barriers to walking on streets where no sidewalks existed, especially by participants living near highways [96–99, 103, 104, 108–111]. A lack of street lighting was also mentioned as a barrier [96, 99, 100, 112].

In the study by Cleland et al., participants described that the connectivity of walking and cycling networks with other destinations positively influenced their PA, as well as flat terrain, short distance, and safety [112].

#### Greenness/natural environment

The rural natural environment, including attractive features like streams, lakes, and mountains, was seen as an asset for leisure-time outdoor PA (e.g., hiking, running, skiing, or fishing) in several studies, including the two studies of high quality [95, 105, 107, 108, 112].

## Discussion

This systematic review aimed to summarize evidence on the relationship between the built and natural environment and PA in adults living in rural areas. 51 quantitative studies and 19 qualitative studies were included in the synthesis. Based on the quantitative studies, we found convincing evidence of positive associations between the availability and accessibility of places for exercise and recreation as well as the overall environment and leisure-time PA. Possible positive associations were found between the overall environment and total and transport-related PA, between greenness/natural environment and total PA, between cycling infrastructure and aesthetics and MVPA, and between pedestrian infrastructure and total walking. A possible negative relationship was found between safety and security and total walking. Qualitative studies complemented and confirmed several environmental facilitators (e.g., facilities for exercise and recreation, sidewalks or streets with low traffic, attractive natural environment) and barriers (e.g., lack of facilities and destinations, lack of sidewalks, speeding traffic and high traffic volumes, lack of street lighting). The findings and their implications are discussed in detail below.

### Environmental characteristics convincingly related to PA

#### Availability and accessibility of places for exercise and recreation

Quantitative findings provide convincing evidence for a positive association between the availability and accessibility of places for exercise and recreation and leisure-time PA in rural areas. Qualitative findings strongly support this association. Facilities such as swimming pools, parks, sports fields, walking trails, or public schools were frequently described as an important factor in PA. This finding is consistent with previous reviews focusing on rural areas [21, 22, 30]. Systematic reviews focusing on general populations or urban areas demonstrated mixed results for the association between the availability of places for exercise and recreation and PA in adults [13, 16, 20, 114]. Studies have shown that rural residents usually encounter fewer opportunities to be physically active than urban residents [76, 87, 115, 116]. The provision of accessible facilities for exercise and recreation might be a promising way to promote leisure-time PA in adults living in rural areas. However, longitudinal studies are needed to confirm this. Where school facilities exist, shared use agreements between schools and community partners are a recommended strategy that has not yet reached its full potential in rural areas [117].

#### Overall environment

Quantitative findings provide convincing evidence for a positive association between the overall environment and leisure-time PA and evidence for a possible association with transport-related and total PA. Most of the variables included in this category were aggregate measures including different constructs, such as pedestrian infrastructure, safety, or the presence of destinations. Therefore, the results indicate that a combination of environmental features is associated with leisure-time PA in rural areas, but it remains unclear which elements of the environment make a difference. A Europe-specific review found convincing evidence of a positive relationship between the overall quality of the environment and total PA [20]. Smith et al. did not include aggregate measures in their systematic review, as it is difficult to determine which components of an aggregate measure are most effective and they are therefore difficult to interpret [118].

### Environmental characteristics possibly related to PA

#### Pedestrian infrastructure

Quantitative studies suggest that there is a possible positive association between pedestrian infrastructure and total walking. Qualitative studies describe that the presence of sidewalks facilitates walking. A lack of sidewalks has been identified as a barrier to PA in rural areas by previous reviews [22, 30]. Frost et al. presented four studies with positive associations between sidewalks and PA and one study with negative associations in older adults [21]. Systematic reviews focusing on general populations or urban areas found sidewalks/walking infrastructure to be positively related to walking for transport and transport-related PA [13, 40]. On the other hand, qualitative studies suggest that sidewalks are not necessary in small rural streets where traffic is limited. This might explain why some studies asking for the presence of sidewalks did not find a significant association with walking.

#### Cycling infrastructure

Quantitative findings provide evidence for a possible positive association between cycling infrastructure and MVPA. This finding is supported by qualitative studies that discussed the importance of safe places for cycling. Cycling infrastructure did not play any role in previous reviews focusing on rural populations [21, 22, 30]. Hansen et al. argued that active transportation was not realistic for many rural residents because of the great distances between destinations [22]. However, the United States 2017 National Household Travel Survey revealed that urban and rural areas had similar prevalence of overall cycling and cycling for exercise [119]. Active travel on longer distances has become more feasible with the emergence of the e-bike [120, 121]. Systematic reviews focusing on general populations or urban areas found bicycle lanes to be positively related to cycling for transport and transport-related PA [13, 40]. Providing safe and sufficiently wide cycling paths that are separated from busy roads might facilitate cycling also in rural areas. This relationship should be addressed by future studies since this systematic review did not identify any quantitative studies examining the relationship between cycling infrastructure and cycling behavior in rural areas.

#### Safety and security

Quantitative studies suggest that there is a possible negative association between safety and security and total walking. Other types of PA (MVPA, leisure-time PA, and transport-related PA) were convincingly unrelated to safety and security. On the contrary, qualitative studies found that perceived pedestrian safety was an important factor in PA. The participants described sidewalks or low-traffic streets as safe for walking. The counterintuitive quantitative results might be explained by a higher awareness of traffic volumes by people who walk more frequently. Besides, safety and security measures were quite heterogeneous (including, for example, perceived traffic volumes, street lighting, and the presence of crosswalks), so that a profound conclusion is hampered. Even more, due to the small number of studies investigating each measure, we were not able to provide more detailed results.

#### Aesthetics

Quantitative findings provide evidence for a possible positive association between aesthetics (interesting things to look at, cleanliness) and MVPA. This finding was not supported by any qualitative study. In addition, aesthetics were convincingly unrelated to leisure-time PA and transport-related PA. Therefore, it remains unclear whether the perceived aesthetics of the environment have any impact on rural adults’ PA. Aesthetics demonstrated a significant positive association with PA in the previous review by Frost et al. [21]. Findings from systematic reviews in urban and general populations are inconsistent [13, 16].

#### Greenness/natural environment

Quantitative findings provide evidence for a possible positive association between objectively measured greenness/natural environment and total PA. Qualitative findings support this association. The participants of several qualitative studies reported that the rural natural environment, including attractive features like streams, lakes, and mountains, supports leisure-time outdoor PA. There is a lack of quantitative measures of the perceived natural environment in rural areas. In the included studies, subjective measures mostly asked for the presence of trees along the streets [41, 43, 76, 89], not resulting in any significant associations with PA. Perceptions of neighborhood tree cover have been described to be positively related to PA in urban areas [122]. However, the results of this review indicate that questionnaires developed to measure urban natural environments may not be valid in rural areas, where there is a greater variety of natural environments. The RALPESS questionnaire, which was developed specifically to measure perceptions of rural environments in the context of physical activity, does not include any perceptions of the natural environment [92]. Hansen et al. stated in their previous review that there was limited literature examining natural active living environments in rural areas and advocated for studying promising ways to link rural residents to these areas and identifying barriers to accessibility [22]. Natural active living environments in rural areas should be addressed by further research.

### Environmental characteristics not related to PA or with inconclusive results

The availability and accessibility of destinations are convincingly unrelated to MVPA. However, MVPA encompasses different types of PA. Qualitative studies indicate that the availability and accessibility of destinations are related to walking. However, only three studies examined associations between destinations and walking, pointing to a possible positive association [57, 76, 79]. More research is needed to establish whether the availability and accessibility of destinations is related to walking in rural areas. Other environmental characteristics (i.e., availability and accessibility of public transport, overall accessibility, density, land use, connectivity, and hilliness) have been examined in too few studies, so we were not able to get any summary results for them.

### Overall discussion of the results

Overall, this systematic review reveals only two environmental characteristics that are convincingly positively related to PA in rural adults. One of them is the overall environment, which can consist of different measures. This result suggests that the built and natural environment in general terms are associated with PA in rural adults. Qualitative studies support this suggestion, as some environmental characteristics are consistently described as facilitators or barriers to PA. However, not all quantitative studies seem to be able to capture the environmental characteristics that are important in rural areas. The majority of studies rely on subjective instruments that have been developed and validated in urban areas, such as the NEWS or other questionnaires, and that might not be suitable for rural areas.

Qualitative studies can help to identify environmental concepts that are not fully covered by quantitative measures. The results of the qualitative studies could be used to further refine measures to quantitatively confirm associations between the built and natural environment and PA.

### Strengths and limitations

First, a strength of this systematic review is the inclusion of quantitative and qualitative studies and thus a more complete synthesis of the current evidence. Second, we stratified the results based on the type of PA as suggested to improve current practice in reviews of the built environment and PA [11].

The included studies have some limitations. All included studies are either cross-sectional studies, ecological studies, or qualitative studies. No longitudinal studies were identified. Cross-sectional and ecological studies cannot contribute to the demonstration of a causal relationship between the built and natural environment and PA and the latter one is prone to an ecological fallacy. Most of the included quantitative studies relied on self-reported measurements of PA, which are a potential source of bias. Not all quantitative studies accounted for possible confounders, such as residential self-selection. Most of the qualitative studies had either medium or low quality and lacked a clearly described and systematic data analysis.

There are also some limitations related to the review method. First, only published journal articles in English language were included, and gray literature and articles in any other language were excluded. Therefore, publication bias cannot be ruled out. Second, the restriction for English-written publications might also have led to an over-representation of studies conducted in the United States (two-thirds of quantitative and almost 90% of qualitative studies). As a result, the conclusions of this review might not generalize to different geographic regions. Third, the review is limited by the databases and search terms employed. Fourth, the review summarizes results from heterogeneous studies applying different definitions of rurality, different spatial units, and different instruments.

## Conclusions

Research investigating the relationship between the built and natural environment and PA behaviors of adults living in rural areas is still limited. There is a need for more high-quality studies in terms of study design, valid and reliable measures of PA, and a broad spectrum of environmental features. Furthermore, we suggest that further research focuses on longitudinal studies, including natural experiments, and conceptually matched associations with specific types of PA. Based on the findings of this systematic review, the provision of places for exercise and recreation, the provision of safe walking infrastructure, and the promotion of nature-based activities are possible strategies that should be considered to address low levels of PA in rural adults.

### Electronic supplementary material

Below is the link to the electronic supplementary material.

**Supplementary Material 1: Additional file 1:** Search strategy [databases and search terms]

**Supplementary Material 2: Additional file 2:** Detailed synthesis of the quantitative studies

**Supplementary Material 3: Additional file 3:** Definitions of rural areas applied in the studies

## Data Availability

The tables and supplementary material contain all data relevant to the results of the systematic review. The data extraction forms are available from the corresponding author upon request.

## Abbreviations

MVPA: Moderate-to vigorous physical activity
PA: Physical activity
PRISMA: Preferred Reporting Items for Systematic Reviews and Meta-analyses
RALA: Rural Active Living Assessment
RALPESS: Rural Active Living Perceived Environmental Support Scale
WHO: World Health Organization
