# Supplementary material for: Built and natural environment correlates of physical activity of adults living in rural areas: a systematic review
Source: Int J Behav Nutr Phys Act. 2024 May 3;21:52. doi: 10.1186/s12966-024-01598-3 (PMC11067138; doi:10.1186/s12966-024-01598-3)
Supplement: Supplementary file 1 — Supplementary Material 1: Additional file 1: Search strategy [databases and search terms] [file 12966_2024_1598_MOESM1_ESM.docx]

**Additional File 1**

**Sensitivity analysis (PUBMED, 28 September 2021)**

| **Keyword(s) rural areas** | **Additional keywords** | **No. of results** | **Relevant without rural*** |
| --- | --- | --- | --- |
| (rural [TIAB] OR “small town” [TIAB] OR “small towns” [TIAB] OR village* [TIAB] OR countryside [TIAB]) | AND (“built environment*” [TIAB] OR “physical environment*” [TIAB] OR “natural environment*” [TIAB] OR “area-level” [TIAB] OR walkability [TIAB] OR bikeability [TIAB] OR “land use*” [TIAB] OR “green space*” [TIAB] OR greenspace* [TIAB] OR “open space*” [TIAB] OR greenness [TIAB] OR “blue space*” [TIAB] OR forest* [TIAB] OR landscape* [TIAB] OR vegetation [TIAB] OR nature [TIAB] OR neighbourhood* [TIAB] OR neighborhood* [TIAB] OR ecological [TIAB] OR infrastructure [TIAB] OR “recreation facilities” [TIAB] OR sidewalk [TIAB] OR park OR “physical attributes” [TIAB] OR “physical characteristics” [TIAB] OR playground [TIAB]) AND (“physical activity” [TIAB] OR sport* [TIAB] OR exercise [TIAB] OR walking [TIAB] OR walk [TIAB] OR cycling [TIAB] OR cycle [TIAB] OR bicycle [TIAB] OR biking [TIAB] OR “active transport” [TIAB] OR “active travel” [TIAB] OR “active commuting” [TIAB ] OR “everyday activities” [TIAB] OR “motorized travel” [TIAB] OR “motorized transport” [TIAB]) AND (determinant [TIAB] OR determinants [TIAB] OR correlate [TIAB] OR correlates [TIAB] OR influence [TIAB] OR influences [TIAB] OR association [TIAB] OR associations [TIAB] OR predictor* [TIAB] OR barrier* [TIAB] OR enabler* [TIAB] OR facilitator* [TIAB]) | 547 |  |
| rural [TIAB] |  | 507 |  |
| village [TIAB] |  | 48 | 0* |
| („small town“ [TIAB] OR „small towns” [TIAB]) |  | 6 | 0* |
| countryside [TIAB] |  | 4 | 0* |
| *We read the titles and abstracts of the results to check whether the alternative keywords identified any additional records that did not include the keyword “rural” in their titles and abstracts. | | | |

**Search strategy (databases and search terms)**

| PUBMED | rural [TIAB] AND (“built environment*” [TIAB] OR “physical environment*” [TIAB] OR “natural environment*” [TIAB] OR “area-level” [TIAB] OR walkability [TIAB] OR bikeability [TIAB] OR “land use*” [TIAB] OR “green space*” [TIAB] OR greenspace* [TIAB] OR “open space*” [TIAB] OR greenness [TIAB] OR “blue space*” [TIAB] OR forest* [TIAB] OR landscape* [TIAB] OR vegetation [TIAB] OR nature [TIAB] OR neighbourhood* [TIAB] OR neighborhood* [TIAB] OR ecological [TIAB] OR infrastructure [TIAB] OR “recreation facilities” [TIAB] OR sidewalk [TIAB] OR park OR “physical attributes” [TIAB] OR “physical characteristics” [TIAB] OR playground [TIAB]) AND (“physical activity” [TIAB] OR sport* [TIAB] OR exercise [TIAB] OR walking [TIAB] OR walk [TIAB] OR cycling [TIAB] OR cycle [TIAB] OR bicycle [TIAB] OR biking [TIAB] OR “active transport” [TIAB] OR “active travel” [TIAB] OR “active commuting” [TIAB ] OR “everyday activities” [TIAB] OR “motorized travel” [TIAB] OR “motorized transport” [TIAB]) AND (determinant [TIAB] OR determinants [TIAB] OR correlate [TIAB] OR correlates [TIAB] OR influence [TIAB] OR influences [TIAB] OR association [TIAB] OR associations [TIAB] OR predictor* [TIAB] OR barrier* [TIAB] OR enabler* [TIAB] OR facilitator* [TIAB]) |
| --- | --- |
| PsycInfo | AB,TI(rural) AND AB,TI(“built environment*” OR “physical environment*” OR “natural environment*” OR “area-level” OR walkability OR bikeability OR “land use*” OR “green space*” OR greenspace* OR “open space*” OR greenness OR “blue space*” OR forest* OR landscape* OR vegetation OR nature OR neighbourhood* OR neighborhood* OR ecological OR infrastructure OR “recreation facilities” OR sidewalk OR park OR “physical attributes” OR “physical characteristics” OR playground) AND AB,TI(“physical activity” OR sport* OR exercise OR walking OR walk OR cycling OR cycle OR bicycle OR biking OR “active transport” OR “active travel” OR “active commuting” OR “everyday activities” OR “motorized travel” OR “motorized transport”) AND AB,TI(determinant OR determinants OR correlate OR correlates OR influence OR influences OR association OR associations OR predictor* OR barrier* OR enabler* OR facilitator*) |
| Web of Science | TS=rural AND TS=(“built environment*” OR “physical environment*” OR “natural environment*” OR “area-level” OR walkability OR bikeability OR “land use*” OR “green space*” OR greenspace* OR “open space*” OR greenness OR “blue space*” OR forest* OR landscape* OR vegetation OR nature OR neighbourhood* OR neighborhood* OR ecological OR infrastructure OR “recreation facilities” OR sidewalk OR park OR “physical attributes” OR “physical characteristics” OR playground) AND TS=(“physical activity” OR sport* OR exercise OR walking OR walk OR cycling OR cycle OR bicycle OR biking OR “active transport” OR “active travel” OR “active commuting” OR “everyday activities” OR “motorized travel” OR “motorized transport”) AND TS=(determinant OR determinants OR correlate OR correlates OR influence OR influences OR association OR associations OR predictor* OR barrier* OR enabler* OR facilitator*) |
| TRID | rural AND (“built environment*” OR “physical environment*” OR “natural environment*” OR “area-level” OR walkability OR bikeability OR “land use*” OR “green space*” OR greenspace* OR “open space*” OR greenness OR “blue space*” OR forest* OR landscape* OR vegetation OR nature OR neighbourhood* OR neighborhood* OR ecological OR infrastructure OR “recreation facilities” OR sidewalk OR park OR “physical attributes” OR “physical characteristics” OR playground) AND  (“physical activity” OR sport* OR exercise OR walking OR walk OR cycling OR cycle OR bicycle OR biking OR “active transport” OR “active travel” OR “active commuting” OR “everyday activities” OR “motorized travel” OR “motorized transport”) AND (determinant OR determinants OR correlate OR correlates OR influence OR influences OR association OR associations OR predictor* OR barrier* OR enabler* OR facilitator*) |
| Engineering Village – GEOBASE & GeoRef | ((rural WN TI) OR (rural WN AB)) AND (((“built environment*” OR “physical environment*” OR “natural environment*” OR “area-level” OR walkability OR bikeability OR “land use*” OR “green space*” OR greenspace* OR “open space*” OR greenness OR “blue space*” OR forest* OR landscape* OR vegetation OR nature OR neighbourhood* OR neighborhood* OR ecological OR infrastructure OR “recreation facilities” OR sidewalk OR park OR “physical attributes” OR “physical characteristics” OR playground) WN TI) OR ((“built environment*” OR “physical environment*” OR “natural environment*” OR “area-level” OR walkability OR bikeability OR “land use*” OR “green space*” OR greenspace* OR “open space*” OR greenness OR “blue space*” OR forest* OR landscape* OR vegetation OR nature OR neighbourhood* OR neighborhood* OR ecological OR infrastructure OR “recreation facilities” OR sidewalk OR park OR “physical attributes” OR “physical characteristics” OR playground) WN AB)) AND (((“physical activity” OR sport* OR exercise OR walking OR walk OR cycling OR cycle OR bicycle OR biking OR “active transport” OR “active travel” OR “active commuting” OR “everyday activities” OR “motorized travel” OR “motorized transport”) WN TI) OR ((“physical activity” OR sport* OR exercise OR walking OR walk OR cycling OR cycle OR bicycle OR biking OR “active transport” OR “active travel” OR “active commuting” OR “everyday activities” OR “motorized travel” OR “motorized transport”) WN AB)) AND (((determinant OR determinants OR correlate OR correlates OR influence OR influences OR association OR associations OR predictor* OR barrier* OR enabler* OR facilitator*) WN TI) OR ((determinant OR determinants OR correlate OR correlates OR influence OR influences OR association OR associations OR predictor* OR barrier* OR enabler* OR facilitator*) WN AB)) |
