# Supplementary material for: Built and natural environment correlates of physical activity of adults living in rural areas: a systematic review
Source: Int J Behav Nutr Phys Act. 2024 May 3;21:52. doi: 10.1186/s12966-024-01598-3 (PMC11067138; doi:10.1186/s12966-024-01598-3)
Supplement: Supplementary file 2 — Supplementary Material 2: Additional file 2: Detailed synthesis of the quantitative studies [file 12966_2024_1598_MOESM2_ESM.docx]

**Additional File 2: Detailed synthesis of the quantitative studies**

**Summary of associations of environmental characteristics with all types of PA, unspecified/total PA, MVPA, leisure-time PA, transport-related PA, and active commuting**

| **Environmental characteristic** | **All types of PA** | **Unspecified/ total PA** | **MVPA** | **Leisure-time PA** | **Sports/ Exercise** | **Transport-related PA** | **Active commuting** |
| --- | --- | --- | --- | --- | --- | --- | --- |
| **Availability and accessibility of destinations** | **0 0** | **N/A** | **0 0** | **N/A** | **N/A** | **N/A** | **N/A** |
| Positive | 6/15 (46, 66, 65, 71, 61, 83) | 0/1 | 0/6 | 1/3 (46) | - | 1/2 (66) (66: many destinations within walking distance of home – agree) | 1/1 (65) |
| Negative | 2/15 (80, 71) | 0/1 | 0/6 | 0/3 | - | 0/2 | 0/1 |
| Non-significant | 14/15 (92, 62, 65, 89, 44, 77, 94, 91, 66, 71, 83, 80, 61, 86) | 1/1 (92) | 6/6 (62, 65, 89, 44, 78, 94) | 2/3 (91, 92) | - | 2/2 (66, 92) (66: many destinations within walking distance of home – strongly agree, count of total non-residential destinations) | 0/1 |
| **Availability and accessibility of places for exercise or recreation** | **+** | **N/A** | **0 0** | **+ +** | **N/A** | **N/A** | **N/A** |
| Positive | 12/27 (56, 65, 89, 49, 59, 46, 48, 66, 83, 61, 63, 64) | 0/1 | 4/16 (56, 65, 89, 49) (65: safe park; 89: insufficiently active vs. inactive; 49: number of hiking trails, number of sports parks) | 3/4 (59, 46, 48) (46: shorter distance to fitness club, park, recreation center, school that allows the public to use their facility for PA, walking trail | 0/1 | 1/2 (66) (11-20 min vs. > 20 min to walk to the nearest trail) | 1/1 (65) (safe park) |
| Negative | 1/27 (59) | 0/1 | 0/16 | 0/4 | 0/1 | 1/2 (59) |  |
| Non-significant | 24/27 (69, 59, 62, 65, 68, 52, 89, 49, 44, 74, 75, 77–79, 94, 84, 46, 57, 85, 66, 80, 61, 63, 64) | 1/1 (69) | 15/16 (59, 62, 65, 68, 52, 89, 49, 44, 74, 75, 77–79, 94, 84) (65: several free or low-cost recreation facilities; 89: sufficiently active vs. inactive; 49: number of exercise facilities, number of parks) | 2/4 (46, 57) (46: many places for PA excluding walking, distance to public swimming pool) | 1/1 (85) | 1/2 (66) (66: 10 min or less vs. > 20 min to walk to the nearest trail, presence of off-road recreation trails and indoor recreation facilities in a 520-m buffer) | 1/1 (65) (65: several free or low-cost recreation facilities) |
| **Availability and accessibility of public transport** | **+** | **N/A** | **N/A** | **N/A** | **N/A** | **N/A** | **N/A** |
| Positive | 5/9 (89, 92, 71, 61, 88) | 1/1 (92) | 1/3 (89) (89: perceived) | 1/1 (92) | - | 0/1 | 0/1 |
| Negative | 1/9 (88) | 0/1 | 0/3 | 0/1 | - | 0/1 | 0/1 |
| Non-significant | 8/9 (65, 89, 92, 94, 83, 61, 86, 88) | 0/1 | 3/3 (65, 89, 94) (89: objective) | 0/1 | - | 1/1 (92) | 1/1 (65) |
| **Overall accessibility** | **0 0** | **N/A** | **N/A** | **N/A** | **N/A** | **N/A** | **N/A** |
| Positive | 0/6 | 0/1 | 0/1 | 0/1 | - | 0/1 | - |
| Negative | 0/6 | 0/1 | 0/1 | 0/1 | - | 0/1 | - |
| Non-significant | 6/6 (67, 93, 55, 72, 73, 86) | 1/1 (67) | 1/1 (93) | 1/1 (67) | - | 1/1 (67) | - |
| **Density** | **?** | **N/A** | **N/A** | **N/A** | **N/A** | **N/A** | **N/A** |
| Positive | 2/7 (65, 64) | 0/1 | 0/3 | - | - | - | 1/1 (65) |
| Negative | 3/7 (50, 86, 64) | 1/1 (50) | 0/3 | - | - | - | 0/1 |
| Non-significant | 4/7 (65, 89, 94, 80) | 0/1 | 3/3 (65, 89, 94) | - | - | - | 0/1 |
| **Mixed land use** | **N/A** | **N/A** | **N/A** | **N/A** | **N/A** | **N/A** | **N/A** |
| Positive | 0/1 | 0/1 | - | 0/1 | - | 0/1 | - |
| Negative | 0/1 | 0/1 | - | 0/1 | - | 0/1 | - |
| Non-significant | 1/1 (67) | 1/1 (67) | - | 1/1 (67) | - | 1/1 (67) | - |
| **Connectivity** | **0 0** | **N/A** | **N/A** | **N/A** | **N/A** | **N/A** | **N/A** |
| Positive | 2/8 (63, 90) | 0/2 | 0/1 | 0/1 | - | 0/1 | - |
| Negative | 2/8 (50, 63) | 1/2 (50) | 0/1 | 0/1 | - | 0/1 | - |
| Non-significant | 7/8 (94, 92, 63, 64, 86, 90, 80) | 1/2 (92) | 1/1 (94) | 1/1 (92) | - | 1/1 (92) | - |
| **Pedestrian infrastructure** | **0 0** | **N/A** | **0 0** | **N/A** | **N/A** | **N/A** | **N/A** |
| Positive | 6/17 (65, 47, 58, 76, 80, 83) | 0/2 | 1/11 (65) | 0/2 | - | 1/3 (47) | 0/1 |
| Negative | 0/17 | 0/2 | 0/11 | 0/2 | - | 0/3 | 0/1 |
| Non-significant | 17/17 (47, 58, 46, 62, 65, 66, 68, 89, 44, 92, 76, 77, 94, 84, 71, 83, 80) | 2/2 (47, 92) | 10/11 (58, 47, 62, 68, 89, 44, 76, 78, 94, 84) | 2/2 (46, 92) | - | 2/3 (66, 92) | 1/1 (65) |
| **Cycling infrastructure** | **+** | **N/A** | **+** | **N/A** | **N/A** | **N/A** | **N/A** |
| Positive | 3/6 (94, 89, 49) | - | 3/5 (89, 49, 94) (89: sufficiently active vs. inactive; 94: maintenance) | - | - | 0/1 | 0/1 |
| Negative | 0/6 | - | 0/5 | - | - | 0/1 | 0/1 |
| Non-significant | 5/6 (89, 65, 66, 44, 94) | - | 4/5 (89, 65, 44, 94) (89: insufficiently active vs. inactive; 94: presence) | - | - | 1/1 (66) | 1/1 (65) |
| **Safety and security** | **0 0** | **?** | **0 0** | **0 0** | **N/A** | **0 0** | **N/A** |
| Positive | 8/26 (47, 50, 46, 67, 92, 71, 80, 61) | 2/5 (47, 50) | 0/14 | 2/7 (46, 67) (46: shoulders on streets; 67: physical security in a 1.00-mile and a 1.62-mile buffer) | - | 1/5 (92) (92: crosswalks) | 0/1 |
| Negative | 7/26 (45, 47, 77, 94, 62, 52, 91) | 1/5 (45) (45: Riverland) | 4/14 (47, 62, 77, 94) (62: fair street lighting in sufficiently vs. insufficiently active and inactive women; 77: very good/good street lighting in sufficiently + insufficiently active vs. inactive women; 94: too much traffic to bicycle) | 0/7 | - | 0/5 | 0/1 |
| Non-significant | 25/26 (45, 67, 92, 58, 59, 52, 89, 44, 74, 77, 79, 84, 94, 62, 46, 87, 91, 47, 66, 65, 72, 71, 73, 80, 61) | 3/5 (45, 67, 92) (45: Yorke Peninsula) | 13/14 (58, 59, 62, 65, 52, 89, 44, 74, 77, 79, 84, 94) (94: too much traffic to walk) | 7/7 (59, 46, 87, 67, 91, 92) (46: safety from traffic; 67: pedestrian safety, physical security in other buffers) | - | 5/5 (59, 47, 66, 67, 92) (92: buffer between sidewalk and street, street lighting, too much traffic to walk) | 1/1 (65) |
| **Aesthetics** | **+** | **N/A** | **+** | **0 0** | **N/A** | **0 0** | **N/A** |
| Positive | 8/16 (54, 67, 89, 44, 84, 91, 72, 73) | 1/3 (54) | 3/5 (89, 44, 84) (44: cleanliness; 89: sufficiently active vs. inactive) | 1/7 (67) (67: in 0.00-miles buffer) | 0/1 | 0/5 | 0/1 |
| Negative | 0/16 | 0/3 | 0/5 | 0/7 | 0/1 | 0/5 | 0/1 |
| Non-significant | 15/16 (67, 92, 65, 89, 44, 94, 53, 54, 46, 87, 91, 51, 66, 72, 73) | 2/3 (67, 92) | 4/5 (65, 89, 44, 94) (44: interesting things to look at, maintenance; 89: insufficiently active vs. inactive) | 7/7 (53, 54, 46, 87, 67, 91, 92) (67: in all other buffers) | 1/1 (51) | 5/5 (53, 54, 66, 67, 92) | 1/1 (65) |
| **Greenness/ natural environment** | **?** | **+** | **N/A** | **N/A** | **N/A** | **N/A** | **N/A** |
| Positive | 4/10 (81, 50, 48, 47) | 2/4 (81, 50) (81: percentage of impervious surface – reversed direction) | 0/2 | 1/3 (48) | - | 1/2 (47) | - |
| Negative | 2/10 (63, 64) | 0/4 | 0/2 | 0/3 | - | 0/2 | - |
| Non-significant | 7/10 (47, 46, 44, 92, 63, 80, 81) | 3/4 (47, 92, 81) (81: greenness) | 2/2 (47, 44) | 2/3 (46, 92) | - | 1/2 (92) | - |
| **Hilliness** | **?** | **N/A** | **N/A** | **N/A** | **N/A** | **N/A** | **N/A** |
| Positive | 2/5 (50, 47) | 1/2 (50) | 1/2 (47) | - | - | 0/1 | - |
| Negative | 0/5 | 0/2 | 0/2 | - | - | 0/1 | - |
| Non-significant | 4/5 (47, 84, 80, 71) | 1/2 (47) | 1/2 (84) | - | - | 1/1 (47) | - |
| **Overall environment** | **+** | **+** | **0 0** | **+ +** | **N/A** | **+** | **N/A** |
| Positive | 11/20 (47, 54, 45, 68, 70, 57, 67, 53, 83, 60, 46) | 3/6 (47, 54, 45) (45: Riverland) | 2/7 (68, 70) (70: indirect effect through self-efficacy and family social support) | 4/6 (54, 67, 57, 46) (57: physical environment and town center walkability; 67: overall built environment in a 1.62-mile buffer) | - | 3/6 (47, 54, 53) (53: ≥ 1 min/week vs. < 1 min/week) | - |
| Negative | 1/20 (93) | 0/6 | 1/7 (93) (93: analysis of covariance) | 0/6 | - | 0/6 | - |
| Non-significant | 16/20 (55, 69, 82, 83, 86, 45, 67, 93, 59, 47, 70, 44, 79, 53, 57, 66) | 4/6 (45, 67, 69, 93) (45: Yorke Peninsula) | 6/7 (47, 59, 70, 44, 79, 93) (70: direct effect; 93: linear regression) | 4/6 (59, 53, 67, 57) (57: area around the home; 67: overall built environment in a 0.00-mile, 0.25-mile, 0.62-mile, and 1.00-mile buffer, path walkability in all buffers) | - | 4/6 (67, 53, 59, 66) (53: ≥ 90 min/week vs. < 90 min/week and ≥ 210 min/week vs. < 210 min/week) | - |
| N/A = not able to get a summary result; + = possible positive relationship; + + = convincing positive relationship; ? = inconclusive | | | | | | | |

**Summary of associations of environmental characteristics with walking and cycling behaviors**

| **Environmental characteristic** | **Total walking** | **Leisure/ recreational walking** | **Walking for transport** | **Walking for commuting purposes** | **Cycling for commuting purposes** | **Total walking and cycling** | **Car use (vs. walking)** |
| --- | --- | --- | --- | --- | --- | --- | --- |
| **Availability and accessibility of destinations** | **N/A** | **N/A** | **N/A** | **N/A** | **N/A** | **N/A** | **N/A** |
| Positive | 1/3 (71) (71: number of schools) | 0/3 | 2/3 (61, 83) (61: a coffee place within a 20-min walk of home in high vs. low walking, post office in high vs. low walking, certain distances to school; 83: cultural places) | - | - | - | 0/1 |
| Negative | 1/3 (71) (71: presence of mall) | 0/3 | 1/3 (80) (80: 1-2 convenience stores vs. 0 stores in 1-km buffer, 1-3 restaurants vs. 0 restaurants) | - | - | - | 0/1 |
| Non-significant | 3/3 (91, 71, 94) (71: presence of religious institution) | 0/3 (92, 80, 83) | 3/3 (61, 80, 83) (61: coffee place within 20-min walk of home in any vs. no walking, post office in any vs. no walking, certain distances to school; 80: > 2 convenience stores vs. 0 stores in 1-km buffer, 4-10 restaurants vs. 0 restaurants, distance to post office, number of ethnic markets, food stores, grocery stores, schools, supermarkets, school hectares; 83: shops) | - | - | - | 1/1 (86) |
| **Availability and accessibility of places for exercise or recreation** | **N/A** | **N/A** | **N/A** | **N/A** | **N/A** | **N/A** | **N/A** |
| Positive | 0/3 | 1/2 (83) | 2/3 (83, 61) (61: trail or path within a 20-min walk of home in any vs. no walking, park or natural recreational area with a 20-min walk of home) | 2/2 (63, 64) | 1/2 (63) (63: all rural tracts) | - | - |
| Negative | 0/3 | 0/2 | 0/3 | 0/2 | 0/2 | - | - |
| Non-significant | 3/3 (52, 69, 94) | 1/2 (80) | 2/3 (80, 61) (61: trail or path within 20-min walk of home in high vs. low walking) | 0/2 | 2/2 (63, 64) (63: small rural tracts) | - | - |
| **Availability and accessibility of public transport** | **N/A** | **N/A** | **N/A** | **N/A** | **N/A** | **N/A** | **N/A** |
| Positive | 1/2 (71) | 0/2 | 2/3 (61, 88) (61: any vs. no walking; 88: accessibility to transportation infrastructure) | - | 1/1 (88) (88: access to ferry, access to paratransit mode) | - | 0/1 |
| Negative | 0/2 | 0/2 | 1/3 (88) (88: access to ferry, access to paratransit mode) | - | 0/1 | - | 0/1 |
| Non-significant | 1/2 (94) | 2/2 (92, 83) | 2/3 (83, 61) (61: high vs. low walking) | - | 1/1 (88) (88: accessibility to transportation infrastructure) | - | 1/1 (86) |
| **Overall accessibility** | **N/A** | **N/A** | **N/A** | **N/A** | **N/A** | **N/A** | **N/A** |
| Positive | 0/2 | 0/1 | 0/1 | - | - | - | 0/1 |
| Negative | 0/2 | 0/1 | 0/1 | - | - | - | 0/1 |
| Non-significant | 2/2 (55, 72) | 1/1 (73) | 1/1 (73) | - | - | - | 1/1 (86) |
| **Density** | **N/A** | **N/A** | **N/A** | **N/A** | **N/A** | **N/A** | **N/A** |
| Positive | 0/1 | 0/1 | 0/1 | 0/1 | 1/1 (64) | - | 1/1 (86) |
| Negative | 0/1 | 0/1 | 0/1 | 1/1 (64) | 0/1 | - | 0/1 |
| Non-significant | 1/1 (94) | 1/1 (80) | 1/1 (80) | 0/1 | 0/1 | - | 0/1 |
| **Land use: cultural** | **N/A** | **N/A** | **N/A** | **N/A** | **N/A** | **N/A** | **N/A** |
| Positive | 1/1 (71) (71: 1.6-4.0% within 1-km buffer) | 0/1 | 0/1 | - | - | - | - |
| Negative | 0/1 | 0/1 | 0/1 | - | - | - | - |
| Non-significant | 1/1 (71) (71: 0.1-1.5% or > 4.1% within 1-km buffer) | 1/1 (80) | 1/1 (80) | - | - | - | - |
| **Land use: manufacturing** | **N/A** | **N/A** | **N/A** | **N/A** | **N/A** | **N/A** | **N/A** |
| Positive | - | 0/1 | 1/2 (61) | - | - | - | - |
| Negative | - | 0/1 | 0/2 | - | - | - | - |
| Non-significant | - | 1/1 (80) | 1/2 (80) | - | - | - | - |
| **Land use: resource production and extraction** | **N/A** | **N/A** | **N/A** | **N/A** | **N/A** | **N/A** | **N/A** |
| Positive | 0/1 | - | 0/1 | - | - | - | - |
| Negative | 1/1 (71) | - | 1/1 (61) (61: any vs. no walking) | - | - | - | - |
| Non-significant | 0/1 | - | 1/1 (61) (61: high vs. low walking) | - | - | - | - |
| **Land use: multifamily, single family, retail, service, transportation** | **N/A** | **N/A** | **N/A** | **N/A** | **N/A** | **N/A** | **N/A** |
| Positive | - | 0/1 | 0/1 | - | - | - | - |
| Negative | - | 0/1 | 0/1 | - | - | - | - |
| Non-significant | - | 1/1 (80) | 1/1 (80) | - | - | - | - |
| **Connectivity** | **N/A** | **N/A** | **N/A** | **N/A** | **N/A** | **N/A** | **N/A** |
| Positive | 0/1 | 0/2 | 0/2 | 1/3 (90) | 1/2 (63) (63: all rural tracts) | - | 0/1 |
| Negative | 0/1 | 0/2 | 0/2 | 1/3 (63) (63: all rural tracts) | 0/2 | - | 0/1 |
| Non-significant | 1/1 (94) | 2/2 (80, 92) | 2/2 (80, 90) | 2/3 (64, 63) (63: small rural tracts) | 2/2 (64, 63) (63: small rural tracts) | - | 1/1 (86) |
| **Pedestrian infrastructure** | **+** | **N/A** | **N/A** | **N/A** | **N/A** | **N/A** | **N/A** |
| Positive | 2/4 (58, 76) (58 + 76: irregular vs. no walking) | 2/3 (80, 83) (83: roads, sidewalks, paths, or trails) | 1/2 (83) (83: roads, sidewalks, paths, or trails) | - | - | - | - |
| Negative | 0/4 | 0/3 | 0/2 | - | - | - | - |
| Non-significant | 4/4 (58, 71, 76, 94) (58 + 76: regular vs. no walking) | 2/3 (92, 83) (83: sidewalks on most streets) | 2/2 (83, 80) (83: sidewalks on most streets) | - | - | - | - |
| **Cycling infrastructure** | **N/A** | **N/A** | **N/A** | **N/A** | **N/A** | **N/A** | **N/A** |
| Positive | 0/1 | - | - | - | - | - | - |
| Negative | 0/1 | - | - | - | - | - | - |
| Non-significant | 1/1 (94) | - | - | - | - | - | - |
| **Safety and security** | **-** | **N/A** | **N/A** | **N/A** | **N/A** | **N/A** | **N/A** |
| Positive | 1/6 (71) (71: crosswalks, low speed of traffic) | 1/3 (80) (80: low speed of traffic) | 1/3 (61) (61: crosswalks, low speed of traffic in high vs. low walking) | - | - | - | - |
| Negative | 3/6 (52, 91, 94) (52: moderate vs. heavy traffic in white adults; 94: too much traffic to walk) |  |  | - | - | - | - |
| Non-significant | 5/6 (58, 52, 71, 72, 94) (52: light vs. heavy traffic in white adults, moderate and light traffic in African American adults; 71: street lighting; 94: too much traffic to bicycle) | 3/3 (73, 92, 80) (80: too much traffic to walk, crosswalks, street lighting) | 3/3 (61, 73, 80) (61: low speed of traffic in any vs. no walking) | - | - | - | - |
| **Aesthetics** | **N/A** | **N/A** | **N/A** | **N/A** | **N/A** | **N/A** | **N/A** |
| Positive | 2/3 (91, 72) (72: aesthetics) | 1/2 (73) (73: aesthetics) | 0/1 | - | - | - | - |
| Negative | 0/3 | 0/2 | 0/1 | - | - | - | - |
| Non-significant | 2/3 (72, 94) (72: low aesthetics) | 2/2 (73, 92) (73: low aesthetics) | 1/1 (73) | - | - | - | - |
| **Greenness/ natural environment** | **N/A** | **N/A** | **N/A** | **N/A** | **N/A** | **N/A** | **N/A** |
| Positive | - | 0/2 | 0/1 | 0/2 | 0/2 | - | - |
| Negative | - | 0/2 | 0/1 | 2/2 (64, 63) | 1/2 (64) | - | - |
| Non-significant | - | 2/2 (92, 80) | 1/1 (80) | 0/2 | 1/2 (63) | - | - |
| **Hilliness** |  |  |  |  |  |  |  |
| Positive | 0/1 | 0/1 | 0/1 | - | - | - | - |
| Negative | 0/1 | 0/1 | 0/1 | - | - | - | - |
| Non-significant | 1/1 (71) | 1/1 (80) | 1/1 (80) | - | - | - | - |
| **Overall environment** | **N/A** | **N/A** | **N/A** | **N/A** | **N/A** | **N/A** | **N/A** |
| Positive | 0/2 | 1/2 (83) | 0/2 | - | - | 1/1 (60) | 0/1 |
| Negative | 0/2 | 0/2 | 0/2 | - | - | 0/1 | 0/1 |
| Non-significant | 2/2 (55, 69) | 1/2 (82) | 2/2 (82, 83) | - | - | 0/1 | 1/1 (86) |
| N/A = not able to get a summary result; + = possible positive relationship; - = possible negative relationship | | | | | | | |

**References cited in the tables**

44. Lo BK, Graham ML, Folta SC, Paul LC, Strogatz D, Nelson ME et al. Examining the associations between walk score, perceived built environment, and physical activity behaviors among women participating in a community-randomized lifestyle change intervention trial: Strong hearts, healthy communities. Int J Environ Res Public Health 2019; 16(5).

45. Dollman J, Hull M, Lewis N, Carroll S, Zarnowiecki D. Regional differences in correlates of daily walking among middle age and older Australian rural adults: Implications for health promotion. Int J Environ Res Public Health 2016; 13(1).

46. Deshpande AD, Baker EA, Lovegreen SL, Brownson RC. Environmental correlates of physical activity among individuals with diabetes in the rural midwest. Diabetes Care 2005; 28(5):1012–8.

47. Chrisman M, Nothwehr F, Janz K, Yang J, Oleson J. Perceived Resources and Environmental Correlates of Domain-Specific Physical Activity in Rural Midwestern Adults. J Phys Act Health 2015; 12(7):948–7.

48. Michimi A, Wimberly MC. Natural Environments, Obesity, and Physical Activity in Nonmetropolitan Areas of the United States. J Rural Health 2012; 28(4):518–527.

49. Kim B, Hyun HS. Associations between social and physical environments, and physical activity in adults from urban and rural regions. Osong Public Health Res Perspect 2018; 9(1):16–24.

50. Valson JS, Kutty VR, Soman B, Jissa VT. Spatial Clusters of Diabetes and Physical Inactivity: Do Neighborhood Characteristics in High and Low Clusters Differ? Asia Pac J Public Health 2019; 31(7):732–21.

51. Liu P, Wang J, Wang X, Nie W, Zhen F. Measuring the association of self-perceived physical and social neighborhood environment with health of Chinese rural residents. Int J Environ Res Public Health 2021; 18(16).

52. Hooker SP, Wilson DK, Griffin SF, Ainsworth BE. Perceptions of environmental supports for physical activity in African American and white adults in a rural county in South Carolina. Prev Chronic Dis 2005; 2(4):A11-A11.

53. Cleland VJ, Ball K, King AC, Crawford D. Do the Individual, Social, and Environmental Correlates of Physical Activity Differ Between Urban and Rural Women? Environ Behav 2012; 56(3):472–84.

54. Cleland V, Sodergren M, Otahal P, Timperio A, Ball K, Crawford D et al. Associations between the perceived environment and physical activity among adults aged 67–76 years: does urban-rural area of residence matter? J Aging Phys Act 2015; 23(1):67–74.

55. Berry NM, Coffee NT, Nolan R, Dollman J, Sugiyama T. Neighbourhood environmental attributes associated with walking in South Australian adults: differences between urban and rural areas. Int J Environ Res Public Health 2017; 14(9).

56. Abildso CG, Daily SM, Meyer MRU, Edwards MB, Jacobs L, McClendon M et al. Environmental factors associated with physical activity in rural U.S. counties. Int J Environ Res Public Health 2021; 18(14).

57. Kegler MC, Gauthreaux N, Hermstad A, Arriola KJ, Mickens A, Ditzel K et al. Inequities in Physical Activity Environments and Leisure-Time Physical Activity in Rural Communities. Prev Chronic Dis 2022; 19:E52.

58. Addy CL, Wilson DK, Kirtland KA, Ainsworth BE, Sharpe P, Kimsey D. Associations of perceived social and physical environmental supports with physical activity and walking behavior. Am J Public Health 2004; 94(3):560–3.

59. Beck AM, Serrano NH, Toler A, Brownson RC. Multilevel correlates of domain-specific physical activity among rural adults - a cross-sectional study. BMC Public Health 2022; 22(1):2162.

60. Chrisman M, Nothwehr F, Yang J, Oleson J. Perceived Correlates of Domain-Specific Physical Activity in Rural Adults in the Midwest. J Rural Health 2014; 30(4):474–8.

61. Doescher MP, Lee C, Berke EM, Adachi-Mejia AM, Lee CK, Stewart O et al. The built environment and utilitarian walking in small U.S. towns. Prev Med 2014; 80:91–6.

62. Eyler AA. Personal, social, and environmental correlates of physical activity in rural Midwestern white women. Am J Prev Med 2003; 25(3):86–92.

63. Fan JX, Wen M, Kowaleski-Jones L. Sociodemographic and Environmental Correlates of Active Commuting in Rural America. J Rural Health 2015; 31(2):187–85.

64. Fan JX, Wen M, Wan N. Built environment and active commuting: rural-urban differences in the U.S. SSM Popul Health 2017; 3:555–53.

65. Fields R, Kaczynski AT, Bopp M, Fallon E. Built environment associations with health behaviors among Hispanics. J Phys Act Health 2013; 10(3):456–54.

66. Grabow ML, Bernardinello M, Bersch AJ, Engelman CD, Martinez-Donate A, Patz JA et al. What Moves Us: Subjective and Objective Predictors of Active Transportation. J Transp Health 2019; 15.

67. Gustat J, Anderson CE, Chukwurah QC, Wallace ME, Broyles ST, Bazzano LA. Cross-sectional associations between the neighborhood built environment and physical activity in a rural setting: the Bogalusa Heart Study. BMC Public Health 2020; 20(1).

68. Haslam A, Taniguchi T, Love C, Jacob T, Cannady TK, Standridge J et al. Perceived Environments and Physical Activity Among American Indian Adults Living in Oklahoma: The THRIVE Study. Prog Community Health Partnersh 2021; 15(3):285–96.

69. Jilcott Pitts SB, Keyserling TC, Johnston LF, Smith TW, McGuirt JT, Evenson KR et al. Associations between neighborhood-level factors related to a healthful lifestyle and dietary intake, physical activity, and support for obesity prevention polices among rural adults. J Community Health 2015; 52(2):287–84.

70. Kegler MC, Swan DW, Alcantara I, Feldman L, Glanz K. The Influence of Rural Home and Neighborhood Environments on Healthy Eating, Physical Activity, and Weight. Prev Sci 2014; 15(1):1–11.

71. Lee C, Lee C, Stewart OT, Carlos HA, Adachi-Mejia A, Berke EM et al. Neighborhood Environments and Utilitarian Walking Among Older vs. Younger Rural Adults. Front Public Health 2021; 9.

72. Li C, Chi G, Jackson R. Perceptions and barriers to walking in the rural South of the United States: The influence of neighborhood built environment on pedestrian behaviors. Urban Des Int 2015; 20(4):267–84.

73. Li C, Chi G, Jackson R. Neighbourhood built environment and walking behaviours: Evidence from the rural American South. Indoor Built Environ 2018; 27(7):950–64.

74. Osuji T, Lovegreen S, Elliott M, Brownson RC. Barriers to physical activity among women in the rural midwest. Women Health 2006; 56(1):53–67.

75. Parks SE, Housemann RA, Brownson RC. Differential correlates of physical activity in urban and rural adults of various socioeconomic backgrounds in the United States. J Epidemiol Community Health 2003; 69(1):29–46.

76. Reed JA, Wilson DK, Ainsworth BE, Bowles H, Mixon G. Perceptions of neighborhood sidewalks on walking and physical activity patterns in a southeastern community in the US. J Phys Act Health 2006; 3(2):255–65.

77. Sanderson BK, Cornell CE, Bittner V, Pulley LV, Kirk K, Yang Y et al. Physical activity patterns among women in rural Alabama. Am J Health Behav 2003; 27(4):311–21.

78. Sanderson BK, Foushee HR, Bittner V, Cornell CE, Stalker V, Shelton S et al. Personal, social, and physical environmental correlates of physical activity in rural African-American women in Alabama. Am J Prev Med 2003; 25(3 Suppl 1):30–7.

79. Serrano N, Beck A, Salvo D, Eyler A, Reis R, Steensma JT et al. Examining the Associations of and Interactions Between Intrapersonal and Perceived Environmental Factors With Objectively Assessed Physical Activity Among Rural Midwestern Adults, USA. Am J Health Promot 2022:8901182221147107.

80. Stewart OT, Moudon AV, Saelens BE, Lee C, Kang B, Doescher MP. Comparing Associations Between the Built Environment and Walking in Rural Small Towns and a Large Metropolitan Area. Environ Behav 2016; 60(1):13–47.

81. Villeneuve PJ, Jerrett M, Su JG, Weichenthal S, Sandler DP. Association of residential greenness with obesity and physical activity in a US cohort of women. Environ Res 2018; 172:503–84.

82. Watson KB, Whitfield GP, Thomas JV, Berrigan D, Fulton JE, Carlson SA. Associations Between the National Walkability Index and Walking Among US Adults — National Health Interview Survey, 2015. Prev Med 2020; 149.

83. Whitfield GP, Carlson SA, Ussery EN, Watson KB, Berrigan D, Fulton JE. National-level environmental perceptions and walking among urban and rural residents: Informing surveillance of walkability. Prev Med 2019; 123:101–8.

84. Wilcox S, Castro C, King AC, Housemann R, Brownson RC. Determinants of leisure time physical activity in rural compared with urban older and ethnically diverse women in the United States. J Epidemiol Community Health 2000; 66(9):678–83.

85. An R, Zheng J. Proximity to an exercise facility and physical activity in China. Southeast Asian J Trop Med Public Health 2014; 57(6):1614–91.

86. Ao Y, Zhang Y, Wang Y, Chen Y, Yang L. Influences of rural built environment on travel mode choice of rural residents: The case of rural Sichuan. J Transp Geogr 2020; 85.

87. Ding D, Sallis JF, Hovell MF, Du J, Zheng M, He H et al. Physical activity and sedentary behaviours among rural adults in suixi, china: a cross-sectional study. Int J Behav Nutr Phys Act 2011; 8.

88. Singh SS, Sarkar B. Transport accessibility and affordability as the determinant of non-motorized commuting in rural India. Transp Policy 2022; 118:101–11.

89. Kamada M, Kitayuguchi J, Inoue S, Kamioka H, Mutoh Y, Shiwaku K. Environmental Correlates of Physical Activity in Driving and non-driving Rural Japanese Women. Prev Med 2009; 61(6):pp 610-616.

90. Koohsari MJ, Sugiyama T, Shibata A, Ishii K, Liao Y, Hanibuchi T et al. Associations of street layout with walking and sedentary behaviors in an urban and a rural area of Japan. Health Place 2017; 57:75–9.

91. Kirby AM, Levesque L, Wabano V, Robertson-Wilson J. Perceived community environment and physical activity involvement in a northern-rural Aboriginal community. Int J Behav Nutr Phys Act 2007; 4(74).

92. Malambo P, Kengne AP, Lambert EV, Villers A de, Puoane T. Association between perceived built environmental attributes and physical activity among adults in South Africa. BMC Public Health 2017; 17.

93. Solbraa AK, Anderssen SA, Holme IM, Kolle E, Hansen BH, Ashe MC. The built environment correlates of objectively measured physical activity in Norwegian adults: A cross-sectional study. J Sport Health Sci 2018; 7(1):19–26.

94. Wallmann B, Bucksch J, Froboese I. The association between physical activity and perceived environment in German adults. Eur J Public Health 2012; 22(4):622–8.
