# Supplementary material for: Built and natural environment correlates of physical activity of adults living in rural areas: a systematic review
Source: Int J Behav Nutr Phys Act. 2024 May 3;21:52. doi: 10.1186/s12966-024-01598-3 (PMC11067138; doi:10.1186/s12966-024-01598-3)
Supplement: Supplementary file 3 — Supplementary Material 3: Additional file 3: Definitions of rural areas applied in the studies [file 12966_2024_1598_MOESM3_ESM.docx]

**Additional File 3**

Definitions of rural areas applied in the included quantitative and qualitative studies

| **Definition of rural** | **Studies** |
| --- | --- |
| **U.S. Bureau of Census (2010)**  **2010 Census Urban and Rural Classification and Urban Area Criteria**  “Rural” encompasses all population, housing, and territory not included within an urban area.  Urban areas are defined as   - Urbanized Areas (UAs) of 50,000 or more people - Urban Clusters (UCs) of at least 2,500 and less than 50,000 people.   U.S. Census Bureau, 2010. 2010 Census Urban and Rural Classification and Urban Area Criteria. URL https://www.census.gov/programs-surveys/geography/guidance/geo-areas/urban-rural/2010-urban-rural.html | **Quantitative studies**  n=8 (15.7%)   - Abildso 2021 - Grabow 2019 - Lee 2021 - Li 2018 - Li 2015 - Stewart 2016 - Watson 2020 - Whitfield 2019   **Qualitative studies**  n=1 (5.3%)   - Gilbert 2019 |
| **U.S. Bureau of Census (2000)**  **2000 Census Urban and Rural Classification**  All territory, population, and housing units located outside of urbanized areas (UAs) and urban clusters (UCs).  Urbanized areas and urban clusters are defined as   - core census block groups or blocks that have a population density of at least 1,000 people per square mile and - surrounding census blocks that have an overall density of at least 500 people per square mile   U.S. Census Bureau, 2000. 2000 Census Urban and Rural Classification. URL https://www.census.gov/programs-surveys/geography/guidance/geo-areas/urban-rural/2000-urban-rural.html | **Quantitative studies**  n=2 (3.9%)   - Eyler 2003 - Parks 2003   **Qualitative studies**  n=1 (5.3%)   - Eyler 2002 |
| **U.S. Bureau of Census (1990)**  **Urban and rural definitions**  Territory, population, and housing units not classified as  urban constitute "rural."  Urban areas are defined as   - Places of 2,500 or more persons incorporated as cities, villages, boroughs (except in Alaska and New York), and towns (except in the six New England States, New York, and Wisconsin), but excluding the rural portions of "extended cities." - Census designated places of 2,500 or more persons. - Other territory, incorporated or unincorporated, included in urbanized areas.   U.S. Census Bureau, 1995. Urban and rural definitions. URL https://www2.census.gov/geo/docs/reference/ua/urdef.txt | **Quantitative studies**  N=1 (2.0%)   - Wilcox 2000   **Qualitative studies**  n=0 |
| **U.S. Department of Agriculture (2013)**  **Rural-Urban Continuum Codes**  The Rural-Urban Continuum Codes distinguish U.S. nonmetropolitan (nonmetro) counties by their degree of urbanization and adjacency to a metro area.  Nonmetropolitan counties:   1. Urban population of 20,000 or more, adjacent to a metro area 2. Urban population of 20,000 or more, not adjacent to a metro area 3. Urban population of 2,500 to 19,999, adjacent to a metro area 4. Urban population of 2,500 to 19,999, not adjacent to a metro area 5. Completely rural or less than 2,500 urban population, adjacent to a metro area 6. Completely rural or less than 2,500 urban population, not adjacent to a metro area   USDA Economic Research Service, 2013. Rural-urban continuum codes. URL https://www.ers.usda.gov/data-products/rural-urban-continuum-codes/ | **Quantitative studies**  n=4 (7.8%)   - Beck 2022 - Fields 2013 (earlier version) - Kegler 2022 - Sanderson 2003b (earlier version)   **Qualitative studies**  n=1 (5.3%)   - MacNell 2022 |
| **U.S. Department of Agriculture (USDA) (2000/2010)**  **Primary Rural-Urban Commuting Areas (RUCA) codes**  The rural-urban commuting area (RUCA) codes classify U.S. census tracts using measures of population density, urbanization, and daily commuting.   1. Micropolitan area core: primary flow within an Urban Cluster of 10,000 to 49,999 (large UC) 2. Micropolitan high commuting: primary flow 30% or more to a large UC 3. Micropolitan low commuting: primary flow 10% to 30% to a large UC 4. Small town core: primary flow within an Urban Cluster of 2,500 to 9,999 (small UC) 5. Small town high commuting: primary flow 30% or more to a small UC 6. Small town low commuting: primary flow 10% to 30% to a small UC 7. Rural areas: primary flow to a tract outside a UA or UC   USDA Economic Research Service, 2010. Rural-Urban Commuting Area Codes. URL https://www.ers.usda.gov/data-products/rural-urban-commuting-area-codes/ | **Quantitative studies**  n=3 (5.9%)   - Fan 2015 (2000) - Fan 2017 (2010) - Lo 2019   **Qualitative studies**  n=1 (5.3%)   - Lo 2017 |
| **Nonmetropolitan counties as defined by the Office of Management and Budget (2008)**  Metropolitan Statistical Areas have at least one urbanized area of 50,000 or more population, plus adjacent territory that has a high degree of social and economic integration with the core as measured by commuting ties.  Office of Management and Budget, 2008. Update of statistical area definitions and guidance on their uses. URL https://www.whitehouse.gov/wp-content/uploads/legacy_drupal_files/omb/bulletins/2001-2008/b08-01.pdf | **Quantitative studies**  n=1 (2.0%)   - Michimi 2012   **Qualitative studies**  n=1 (5.3%)  Kaiser 2010 |
| **Australian Bureau of Statistics (2011)**  **Australian Statistical Geography Standard (ASGS)**  **Remoteness Structure**  The Remoteness Structure divides each state and territory into several regions based on their relative access to services. The delimitation criteria for Remoteness Areas are based on the Accessibility/Remoteness Index of Australia (ARIA+) which measures the remoteness of a point based on the physical road distance to the nearest Urban Centre in each of five size classes.   1. Outer Regional Australia: ARIA+ greater than 2.4 and less than or equal to 5.92 2. Remote Australia: ARIA+ greater than 5.92 and less than or equal to 10.53 3. Very Remote Australia: ARIA+ greater than 10.53   Australian Bureau of Statistics, 2011.  Australian Statistical Geography Standard (ASGS) Remoteness Structure URL https://www.abs.gov.au/AUSSTATS/abs@.nsf/allprimarymainfeatures/17A7A350F48DE42ACA258251000C8CA0?opendocument | n=1 qualitative study   - Cleland 2015b |
| Areas falling outside metropolitan Melbourne and outside a 25 km radius of the regional cities (defined as having a population > 20,000) | n=2 quantitative studies   - Cleland 2012 - Cleland 2015a |
| Post codes outside of the capital city region | n=1 quantitative study   - Berry 2017 |
| County with no principal city with a population of over 50,000 people | n=1 quantitative study   - Chrisman 2014   n=1 qualitative study   - Chrisman 2015a |
| Communities with 1000 or fewer people and no community of more than 2500 people within a 15-mile radius | n=1 qualitative study   - Gangeness 2010 |
| Communities with a population of fewer than 15,000 people | n=1 qualitative study   - Seguin 2014 |
| Small towns (≥ 10,000 inhabitants) | n=1 quantitative study   - Doescher 2014 |
| Low population density (5.9 inhabitants/km²) | n=1 quantitative study   - Solbraa 2018 |
| Lower residential density (375-506 inhabitants per km²) | n=1 quantitative study   - Wallmann 2012 |
| Participants indicated whether they were in urban, suburban, rural or in a small town | n=1 quantitative study   - Villeneuve 2018 |
| Not reported | **Quantitative studies**  n=24 (47.1%)   - Addy 2004 - An 2014 - Ao 2020 - Chrisman 2015b - Deshpande 2005 - Ding 2011 - Dollman 2016 - Gustat 2020 - Haslam 2021 - Hooker 2005 - Jilcott Pitts 2015 - Kamada 2009 - Kegler 2014 - Kim 2018 - Kirby 2007 - Koohsari 2017 - Liu 2021 - Malambo 2017 - Osuji 2006 - Reed 2006 - Sanderson 2003a - Serrano 2022 - Singh 2022 - Valson 2019   **Qualitative studies**  n=10 (52.6%)   - Evenson 2002 - Jahns 2014 - Jones 2021 - Kegler 2008 - Maley 2010 - Medagama 2018 - Peterson 2013 - Sanderson 2002 - Thompson 2002 - Whaley 2008 |
